# Comprehensive Characterization of Volatile Flavor Compounds in *Thamnaconus modestus* Under Different Thermal Processing Methods: A Multi-Instrumental Flavoromics Approach

**DOI:** 10.3390/foods15081352

**Published:** 2026-04-13

**Authors:** Qinmei Fang, Ling Ke, Li Bian, Hongshu Chi, Ximin Qiu, Yongcong Chen, Shuigen Li, Siqing Chen, Shaohua Shi

**Affiliations:** 1 Biotechnology Research Institute, Fujian Academy of Agricultural Sciences, Fuzhou 350011, China; 2 State Key Laboratory of Mariculture Biobreeding and Sustainable Goods, Yellow Sea Fisheries Research Institute, Chinese Academy of Fishery Sciences, Qingdao 266071, China; 3 Fujian Fisheries Technology Extension Center, Fuzhou 350011, China

**Keywords:** *Thamnaconus modestus*, thermal processing, volatile flavor compounds, HS-SPME-GC-MS, GC-IMS, odor activity value (OAV), key aroma compounds, flavoromics

## Abstract

*Thamnaconus modestus* (black scraper) is an economically important fish species in Chinese coastal fisheries, yet its pronounced fishy off-odor, primarily attributed to sulfur-containing compounds and trimethylamine (TMA), severely limits consumer acceptance and product diversification. However, a systematic investigation into how different thermal processing methods affect its volatile flavor profile is lacking. This study employed an integrated multi-instrumental flavoromics platform combining sensory evaluation, electronic nose (E-nose), electronic tongue (E-tongue), gas chromatography–ion mobility spectrometry (GC-IMS), and headspace solid-phase microextraction gas chromatography–mass spectrometry (HS-SPME-GC-MS), coupled with chemometric analysis, to systematically characterize the aroma variations of *T. modestus* subjected to steaming, boiling, deep-frying, and roasting treatments compared with raw samples. A total of 62 (GC-IMS) and 129 (GC-MS) volatile compounds were identified, from which 78 characteristic markers (VIP > 1) and 45 key odorants (OAV ≥ 1) were screened. Thermal processing markedly reduced sulfur-containing compounds and TMA concentrations (raw >> steamed ≈ boiled >> deep-fried > roasted) while promoting lipid oxidation- and Maillard reaction-derived aldehydes and furans. Two distinct flavor modulation patterns were revealed: moist-heat methods (steaming, boiling) generated grassy/fatty notes through moderate lipid oxidation, whereas dry-heat methods (deep-frying, roasting) produced characteristic roasted/nutty notes via synergistic activation of Strecker degradation and Maillard reaction. These findings provide scientific evidence for precise flavor quality control and diversified processing optimization of *T. modestus* products.

## 1. Introduction

*Thamnaconus modestus*, commonly known as black scraper or filefish, belongs to the family Monacanthidae and represents an economically important demersal fish species widely distributed in the East China Sea, Yellow Sea, and adjacent waters of Japan and Korea [1,2]. This species is characterized by high protein and low fat content, with muscle tissue rich in PUFAs including EPA and DHA, conferring considerable nutritional and health benefits [2,3]. Owing to its firm texture and minimal intramuscular bones, *T. modestus* has gained increasing popularity in consumer markets and aquatic processing industries [1]. Previous studies have primarily focused on fishery resource assessment [1], nutritional composition [2], and aquaculture biology [4,5], while research on its volatile flavor chemistry remains extremely limited. Among deep-processed products, Zhoushan roasted fish fillet represents the most iconic product, with traditional processing involving slicing, curing, drying, and final roasting at 180–220 °C [6,7]. During thermal processing, PUFAs undergo oxidative degradation, free amino acids participate in Strecker degradation, and reducing sugars generate diverse volatile compounds through Maillard reaction, including aldehydes (hexanal, nonanal), alcohols (1-octen-3-ol), and heterocyclic compounds (2-pentylfuran), collectively constituting the characteristic aroma of processed fish products [8,9,10]. However, fresh *T. modestus* muscle contains relatively high concentrations of TMA and dimethyl sulfide, imparting pronounced fishy off-odor that compromises sensory quality [11,12], largely explaining the current industry’s over-reliance on a single roasting method and constraining product diversification.

Thermal processing plays a crucial role in improving aroma characteristics and reducing undesirable off-odors in aquatic products, as elevated temperatures alter multiple metabolic pathways including oxidative degradation of PUFAs, Strecker degradation of amino acids, Maillard reaction, caramelization, and thermal dissociation of sulfur-containing compounds [8,13,14]. However, existing research on thermal processing-induced volatile changes in aquatic products has predominantly focused on species such as tilapia (*Oreochromis niloticus*) [10], crucian carp (*Carassius auratus*) [15], squid (*Illex argentinus*) [16], and surimi products [17], while systematic investigations on *T. modestus* remain scarce. Although different heating methods—moist-heat (steaming, boiling) and dry-heat (deep-frying, roasting)—generate volatile compounds with distinct compositions through differential activation of lipid oxidation and Maillard reaction [14,18], no study has yet systematically investigated these effects on *T. modestus* [1,2,4,5]. This gap encompasses three specific deficiencies: (1) the volatile compound profile of *T. modestus* has never been characterized using complementary GC-IMS and GC-MS platforms; (2) the differential effects of moist-heat versus dry-heat processing on volatile composition and key odorants remain unknown; and (3) the underlying metabolic pathways responsible for processing-specific aroma transformation have not been elucidated.

To address these gaps, a multi-platform flavoromics framework—defined as a systematic methodological approach for comprehensively analyzing volatile compounds and identifying key aroma contributors [19]—was employed. Sensory evaluation provides direct aroma characterization [20], while E-nose and E-tongue enable rapid discrimination of odor and taste fingerprints [21]. HS-SPME-GC-MS provides detailed qualitative and semi-quantitative volatile identification [9,10,20], whereas GC-IMS offers complementary rapid fingerprint visualization under atmospheric pressure [22,23,24]. Combined use of these platforms yields more comprehensive aroma profiles than either technique alone [10,25,26], yet no study has applied this integrated strategy to characterize volatile flavor compounds of *T. modestus* under different thermal processing methods.

Therefore, this study systematically investigated aroma attributes and volatile compound differences in *T. modestus* muscle subjected to five treatments: raw (control), steamed, boiled, deep-fried, and roasted. Chemometric methods including PCA, hierarchical clustering, and OPLS-DA were applied to screen characteristic marker compounds [27,28], key aroma contributors were identified through OAV calculation [29], and metabolic formation pathways were inferred to elucidate flavor formation mechanisms. We hypothesized that different thermal processing methods would differentially alter the volatile flavor profiles through distinct activation of lipid oxidation, Strecker degradation, and Maillard reaction pathways, with dry-heat methods generating more diverse and intense aroma compounds than moist-heat methods due to higher processing temperatures. Specifically, this study aimed to (1) elucidate how moist-heat and dry-heat processing methods differentially affect volatile compound composition, key odorant profiles, and sensory characteristics of *T. modestus*; (2) identify the metabolic pathways primarily responsible for the processing-specific aroma transformations; and (3) quantify the relationships between key aroma compounds and sensory attributes across processing methods. Understanding these flavor transformations will provide a scientific basis for diversifying *T. modestus* products—from simple roasted fillets to steamed, boiled, or fried offerings with tailored flavor profiles—thereby supporting evidence-based processing optimization and flavor quality control.

## 2. Materials and Methods

### 2.1. Experimental Materials and Chemical Reagents

Fresh *Thamnaconus modestus* were purchased from Zhoushan International Aquatic Products City, Zhoushan, China. Fish specimens measured 22.5 ± 3.0 cm total length and 150.0 ± 25.0 g body weight, all from the same batch of live-caught individuals. All samples were transported to the laboratory within 12 h post-purchase via cold chain (0–4 °C with ice packs) and immediately pretreated. n-Alkane standards (C7–C40) were purchased from Sigma-Aldrich (St. Louis, MO, USA). Internal standard 2-methyl-3-heptanone (purity ≥ 99%) was purchased from Aladdin Biochemical Technology Co., Ltd. (Shanghai, China). Volatile compound standards for absolute quantification (including hexanal, nonanal, 1-octen-3-ol, (E)-2-nonenal, (E,E)-2,4-decadienal, benzaldehyde, 2-methylpyrazine, 2-pentylfuran, 3-methylbutanal, 2-heptanone; all purity ≥ 97%) were purchased from Shanghai Yuanye Bio-Technology Co., Ltd. (Shanghai, China). The solid-phase microextraction (SPME) manual holder and 50/30 μm DVB/CAR/PDMS fiber assembly were purchased from Supelco (Bellefonte, PA, USA). Soybean oil was purchased from Shandong Luhua Group Co., Ltd. (Yantai, China). All other chemical reagents were of analytical grade.

### 2.2. Sample Preparation and Processing Treatments

Fresh *T. modestus* were headed, skinned, and eviscerated, and bilateral dorsal muscles were harvested and trimmed into uniform fish blocks (approximately 50 ± 2 g each). After thorough mixing, samples were randomly assigned to five groups: raw, steamed, boiled, deep-fried, and roasted. Processing parameters for each group were optimized based on previous studies [6,7] and preliminary experiments. Specifically, in the raw group, fish blocks received no thermal treatment and were immediately stored at 4 °C as the control. In the steamed group, approximately 50 g fish blocks were placed on the steamer rack of an electric steamer (Model DZG-A80, Joyoung Co., Ltd., Jinan, China) and steamed at 100 °C for 15 min, with the core temperature reaching ≥85 °C as the endpoint criterion (monitored in real time using a needle-type thermocouple thermometer). In the boiled group, approximately 50 g fish blocks were immersed in boiling distilled water (100 °C; solid-to-liquid ratio 1:5, *w*/*v*) and maintained at a gentle boil for 15 min until the core temperature reached 85 °C. In the deep-fried group, soybean oil was used as the frying medium in a temperature-controlled electric fryer (Model KJ-800, Zhejiang SUPOR Co., Ltd., Hangzhou, China) with oil temperature precisely maintained at 170 ± 2 °C; approximately 50 g fish blocks were fully submerged in hot oil and fried for 5 min, then immediately removed and drained on a stainless steel rack for 30 s. In the roasted group, approximately 50 g fish blocks were placed on the middle rack of an electric oven (Model K43 Pro, Changdi Electrical Appliance Co., Ltd., Foshan, China) and roasted at 200 °C (top and bottom heat) for 15 min, with turning once midway to ensure uniform heating. All thermally processed samples were cooled to 25 ± 2 °C at room temperature after treatment, then immediately subjected to subsequent analysis or stored at −80 °C (Thermo Fisher Scientific, Waltham, MA, USA) until analysis.

### 2.3. Physicochemical Analysis

Moisture content, crude protein content, crude fat content, total volatile basic nitrogen (TVB-N), pH, and thiobarbituric acid reactive substances (TBARS) were determined according to AOAC official methods [30] combined with relevant literature protocols. Moisture content was determined by direct drying (105 °C to constant weight), calculated as the percentage mass difference before and after drying (g/100 g). Crude protein content was determined by the Kjeldahl method using a nitrogen-to-protein conversion factor of 6.25 (g/100 g). Crude fat content was determined by Soxhlet extraction using petroleum ether as the solvent (g/100 g). TVB-N was determined by semi-micro distillation [31] and expressed as mg N/100 g. pH was measured directly in fish muscle homogenate (1:10, *w*/*v*, in deionized water) using a calibrated pH meter. TBARS was determined by thiobarbituric acid colorimetric method and expressed as mg MDA/kg to evaluate the degree of lipid oxidation. All parameters were calculated on a wet weight basis, with three independent replicates per group (n = 3), and results expressed as mean ± standard deviation.

### 2.4. Sensory Evaluation

Sensory evaluation was conducted following Yu et al. [20] with minor modifications. The panel consisted of 12 screened assessors (6 males and 6 females, aged 22–35 years, all graduate students in food science-related fields). All panelists completed a two-week systematic training program covering recognition, classification, and intensity scaling exercises for typical aroma attributes to establish a unified flavor descriptor vocabulary. Formal evaluation employed quantitative descriptive analysis (QDA). The evaluated attributes included fishy odor, fatty aroma, plant aroma, roasted aroma, and sweet aroma. Each attribute was rated on a continuous 0–10 scale (0 = not detected, 10 = extremely strong). Approximately 10 g samples were presented in 30 mL plastic cups labeled with random three-digit codes and served to assessors in randomized order. All evaluations were conducted in individual booths at controlled temperature (25 ± 1 °C), with assessors rinsing their mouths with purified water between samples to eliminate carryover effects. Three independent evaluations (n = 3) were performed for each sample, with each evaluation representing the mean score from all 12 assessors. Results are presented as radar charts.

### 2.5. Electronic Nose Analysis

E-nose analysis was performed using a PEN3.5 portable electronic nose system (Airsense Analytics GmbH, Schwerin, Germany) [20,32]. The core component comprises a sensor array of 10 metal oxide semiconductor (MOS) gas sensors with differential selective responses to various volatile compound types, as follows: W1C (sensitive to aromatic compounds), W5S (broad sensitivity to nitrogen oxides), W3C (sensitive to ammonia and aromatic amines), W6S (sensitive to hydrides), W5C (sensitive to short-chain alkanes and aromatics), W1S (broad sensitivity to methane and other alkanes), W1W (sensitive to organic sulfides and terpenes), W2W (sensitive to alcohols, ethers, aldehydes, and ketones), W2S (sensitive to alcohols and some aromatic compounds), and W3S (sensitive to long-chain alkanes). Approximately 4 g samples were accurately weighed into 20 mL headspace vials, sealed, and equilibrated at 40 °C for 15 min in a water bath. Headspace volatiles were carried into the sensor chamber by ultra-pure air at 150 mL/min. Pre-sampling time was set at 10 s, detection time at 150 s, and sensor cleaning time between measurements at 180 s. Sensor responses were expressed as relative resistance change ratios (R/R_0_), where R_0_ represents baseline resistance in clean carrier gas and R represents steady-state resistance upon exposure to sample volatiles. Steady-state response means from the 48–52 s time window were extracted for subsequent data analysis, including sensor response radar plots, PCA score plots, and LDA discrimination plots. Each sample was analyzed independently three times.

### 2.6. Electronic Tongue Analysis

E-tongue analysis was performed using an SA402B taste sensing system (Insent Inc., Atsugi, Kanagawa, Japan) [33]. This system simulates human taste perception based on electrochemical responses of lipid/polymer membrane sensors and is equipped with the following artificial lipid membrane sensors: AAE sensor (umami), CT0 sensor (saltiness), CA0 sensor (sourness), C00 sensor (bitterness), and AE1 sensor (astringency). Additionally, the system evaluates aftertaste indices by measuring the change in membrane potential caused by adsorption (CPA), including aftertaste-A (richness/umami aftertaste), aftertaste-B (bitterness aftertaste), and aftertaste-C (astringency aftertaste). Reference solution was prepared from 30 mmol/L potassium chloride and 0.3 mmol/L tartaric acid in aqueous solution [33,34].

Sample preparation was as follows: approximately 10 g fish muscle was homogenized with 50 mL deionized water using a high-speed homogenizer (Model T18 digital ULTRA-TURRAX, IKA-Werke GmbH, Staufen, Germany) at 12,000 rpm for 2 min, then centrifuged at 10,000× *g* for 15 min at 4 °C. The supernatant was filtered through a 0.45 μm membrane filter before analysis. Four parallel measurement cycles were performed per sample; to eliminate sensor memory effects from initial contact, the first measurement was discarded and the mean of the subsequent three measurements was used as the final result [34]. E-tongue data were also subjected to PCA and LDA.

### 2.7. GC-IMS Analysis

GC-IMS analysis was performed using a FlavourSpec^®^ flavor analyzer (Gesellschaft für Analytische Sensorsysteme mbH, G.A.S., Dortmund, Germany), comprising an MXT-WAX polar capillary column (15 m × 0.53 mm i.d., 1.0 μm film thickness), IMS detector, and automatic headspace sampler. The analytical method followed Zhao et al. [35] with minor modifications. Approximately 2 g of sample was accurately weighed into 20 mL headspace vials and incubated at 60 °C with 500 rpm oscillation for 15 min. Subsequently, 500 μL headspace gas was automatically injected into the column. Column temperature was maintained at 60 °C; drift tube temperature was set at 45 °C with drift gas (high-purity nitrogen) flow rate of 150 mL/min. Carrier gas was high-purity nitrogen (purity ≥ 99.999%) with the following flow rate gradient program: 2 mL/min for 2 min → linear increase to 10 mL/min for 3 min → increase to 50 mL/min for 5 min → increase to 110 mL/min for 5 min → increase to 150 mL/min for 15 min. Qualitative analysis was based on dual matching of retention time and drift time against the built-in NIST and IMS databases using GC × IMS Library Search software (Version 0.4.10, G.A.S., Dortmund, Germany). Signal intensities (expressed as peak volumes) of volatile compounds were used for semi-quantitative comparison. Three-dimensional spectra, two-dimensional topographic views, and Gallery Plot fingerprint profiles were generated using LAV software (Version 2.2.1, G.A.S., Dortmund, Germany). Each sample was analyzed independently three times.

### 2.8. HS-SPME-GC-MS Analysis

#### 2.8.1. Volatile Compound Extraction

The headspace SPME procedure followed Yu et al. [20] with minor modifications. Approximately 5 g fish muscle and 50 μL internal standard solution (2-methyl-3-heptanone, 1000 μg/L) were transferred to a 20 mL headspace vial (22.5 mm × 75.5 mm), with 1 g NaCl added to enhance salting-out efficiency of volatile compounds. After equilibration at 60 °C for 5 min in a water bath, a DVB/CAR/PDMS fiber (50/30 μm, Supelco, Bellefonte, PA, USA) pre-conditioned at 250 °C for 30 min was inserted into the headspace and extraction was performed at 60 °C with 250 rpm magnetic stirring for 40 min.

#### 2.8.2. Chromatographic–Mass Spectrometric Conditions

GC-MS analysis was performed using a 7890B gas chromatograph coupled with a 5977A mass spectrometer (Agilent Technologies, Santa Clara, CA, USA), equipped with an HP-INNOWAX capillary column (30 m × 0.25 mm i.d., 0.25 μm film thickness; Agilent Technologies, USA). The fiber was thermally desorbed at 250 °C in the GC inlet for 5 min in splitless mode. The oven temperature program was initial 40 °C held for 3 min → ramped at 4 °C/min to 120 °C and held for 2 min → ramped at 6 °C/min to 230 °C and held for 5 min. Carrier gas was high-purity helium (purity ≥ 99.999%) at constant flow of 1.0 mL/min. Mass spectrometric conditions: electron ionization (EI) source at 70 eV, ion source temperature 230 °C, quadrupole temperature 150 °C, transfer line temperature 250 °C, mass scan range *m*/*z* 35–500, full scan mode. Each sample was analyzed independently three times.

#### 2.8.3. Qualitative and Quantitative Analysis and OAV Calculation

Volatile compound identification was based on the combined use of two complementary methods: (1) retention index (RI) comparison—experimental linear RIs were calculated by analyzing C7–C40 n-alkane standard mixtures under identical chromatographic conditions and compared with literature RI values for equivalent column types from the NIST Chemistry WebBook (https://webbook.nist.gov/chemistry/, accessed on 15 December 2025), with deviations within ±20 considered as matches; (2) mass spectral library searching—experimental mass spectra were matched against the NIST 17 mass spectral database, with compounds confirmed when both forward match (SI) and reverse match (RSI) scores were ≥80. For key aroma compounds (including hexanal, nonanal, 1-octen-3-ol, (E)-2-nonenal, (E,E)-2,4-decadienal, benzaldehyde, 2-methylpyrazine, 3-methylbutanal, 2-pentylfuran, 2-heptanone), absolute quantification was performed using the external standard method: standard curves were established by analyzing six concentration gradient standard solutions spiked with equivalent internal standard (2-methyl-3-heptanone) under identical SPME-GC-MS conditions, with compound peak areas plotted against concentrations. All standard curves exhibited linear correlation coefficients R^2^ ≥ 0.995. Remaining compounds were semi-quantified using the internal standard method by calculating approximate concentrations from the ratio of compound peak area to internal standard peak area.

OAV was calculated as the ratio of compound concentration (C, μg/kg) in the sample to its odor threshold (T, μg/kg) in aqueous solution. Threshold values were primarily referenced from Czerny et al. [36] and van Gemert [37]. Compounds with OAV ≥ 1 were identified as key aroma-active compounds with significant contributions to overall aroma.

### 2.9. Statistical Analysis

All experiments were performed with three independent biological replicates (n = 3), consistent with the standard experimental design widely adopted in HS-SPME-GC-MS and GC-IMS volatile compound analysis of thermally processed aquatic products, where n = 3 biological replicates per treatment group has been established as the field norm [10,16,20,24,25]. This sample size is sufficient for parametric statistical tests including one-way ANOVA when combined with post hoc multiple comparisons, and has been shown to reliably detect large effect sizes (Cohen’s d > 0.8) typical of thermal processing-induced volatile changes [10,16]. Data are expressed as mean ± standard deviation (SD). The potential limitations of this sample size on statistical power and generalizability are acknowledged in Section 4. One-way analysis of variance (ANOVA) was performed using SPSS 26.0 software (IBM Corp., Armonk, NY, USA), with Duncan’s test for multiple comparisons at a significance level of *p* < 0.05. Radar charts, stacked bar charts, and bubble plots were generated using Origin 2022 software (OriginLab Corp., Northampton, MA, USA). PCA and OPLS-DA were performed using SIMCA 14.1 software (Sartorius Stedim Data Analytics AB, Umeå, Sweden), with model quality assessed by the goodness of fit (R^2^X, R^2^Y) and predictive ability (Q^2^), and model robustness and validity verified through 200-fold permutation tests. LDA was used for supervised classification of E-nose and E-tongue data. Three-dimensional spectra, two-dimensional topographic views, and Gallery Plot fingerprint profiles for GC-IMS data were generated using LAV software (G.A.S., Dortmund, Germany). Venn diagrams were plotted using the Python 3.8.1 matplotlib_venn library. Hierarchical clustering heatmaps were generated using Ward’s method with Euclidean distance metrics via Python scipy and matplotlib libraries. Pearson correlation analysis between key aroma compounds and sensory attributes was performed using the Python scipy.stats library. Sankey diagrams illustrating associations between key aroma compounds and processing methods were constructed using the Python pyecharts library.

## 3. Results and Discussion

### 3.1. Effects of Different Processing Methods on Physicochemical Properties of T. modestus Muscle

**Figure 1:**
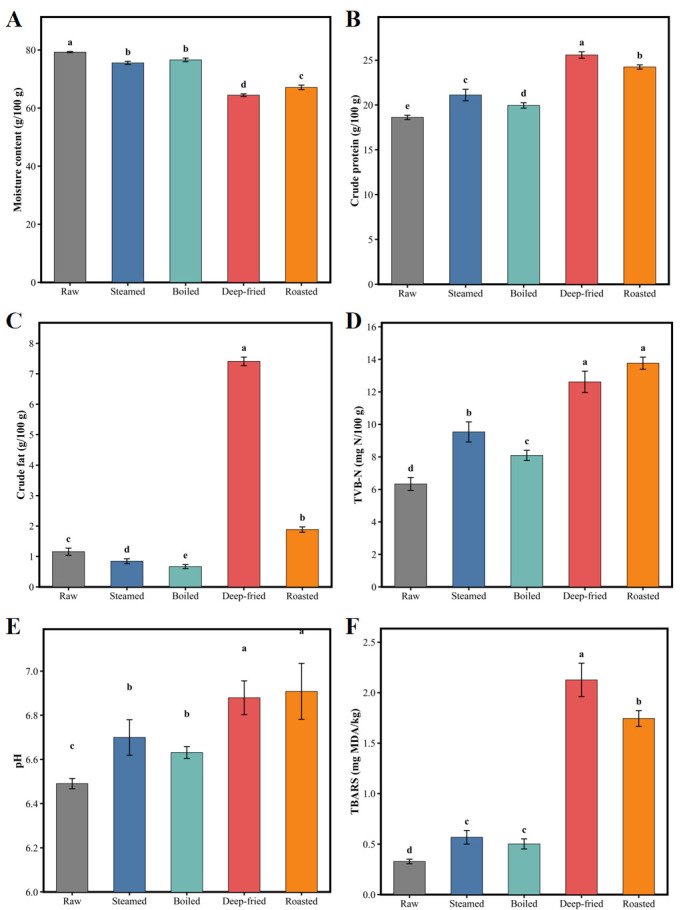
Physicochemical properties of *Thamnaconus modestus* subjected to different processing methods. (**A**) Moisture content; (**B**) crude protein content; (**C**) crude fat content; (**D**) total volatile basic nitrogen (TVB-N) content; (**E**) pH value; (**F**) thiobarbituric acid reactive substance (TBARS) value.

Moisture, crude protein, crude fat, TVB-N, pH, and TBARS values of *T. modestus* muscle under different processing methods are shown in Figure 1. One-way ANOVA revealed significant differences (*p* < 0.05) in all six physicochemical parameters among the five groups, indicating that thermal processing markedly affected the chemical composition and quality characteristics of *T. modestus* muscle. The raw group exhibited moisture content of 79.27 g/100 g, crude protein of 18.61 g/100 g, crude fat of 1.16 g/100 g, TVB-N of 6.33 mg N/100 g, pH of 6.49, and TBARS of 0.33 mg MDA/kg, consistent with reported nutritional profiles of low-fat marine filefish species [2,16,38].

Regarding moisture content (Figure 1A), all thermally processed groups exhibited markedly lower values than the raw group (*p* < 0.05), following the order raw (79.27 g/100 g) > boiled (76.59 g/100 g) > steamed (75.56 g/100 g) > roasted (67.12 g/100 g) > deep-fried (64.43 g/100 g). Deep-fried and roasted groups exhibited the most pronounced moisture loss (18.7% and 15.3% reduction, respectively), attributable to intense protein denaturation and myofibrillar contraction under high-temperature dry-heat conditions (170 °C for frying, 200 °C for roasting), which expelled immobilized water from interfibrillar spaces [8,16]. Steamed and boiled groups showed moderate moisture reduction (3.4–4.7%), as the steam or water medium partially compensated for evaporative loss [7,16], consistent with patterns reported in squid [16] and Spanish mackerel [39].

For crude protein content (Figure 1B), all thermally processed groups exhibited considerably higher values than the raw group (*p* < 0.05): deep-fried (25.57 g/100 g) > roasted (24.24 g/100 g) > steamed (21.11 g/100 g) > boiled (19.95 g/100 g) > raw (18.61 g/100 g). These increases primarily reflect a concentration effect due to moisture loss rather than protein synthesis, consistent with wet weight-based calculations [16,39]. The lower protein content in the boiled group compared to the steamed group can be attributed to leaching of water-soluble sarcoplasmic proteins into the cooking liquid [11,16].

Crude fat content (Figure 1C) showed divergent trends: moist-heat methods decreased fat (0.67–0.85 g/100 g) due to lipid loss via cooking medium, while the deep-fried group showed a dramatic increase (7.41 g/100 g, 540% increase) from oil absorption as moisture evaporated and oil penetrated microscopic pores via capillary action [13,16]. The roasted group showed moderate increase (1.88 g/100 g) attributable to concentration effects. Detailed values for all groups are provided in Figure 1C.

TVB-N reflects the degree of protein degradation, quantified as total volatile nitrogenous bases including TMA, dimethylamine, and ammonia [11,31] (Figure 1D). TVB-N increased markedly after thermal processing (*p* < 0.05), following the order roasted > deep-fried > steamed > boiled > raw (Figure 1D). Dry-heat groups showed 99–117% increases due to accelerated protein thermal degradation and amino acid deamination at 170–200 °C, while moist-heat groups showed milder increases at 100 °C [31].

pH values (Figure 1E) increased notably (*p* < 0.05) following the same roasted > deep-fried > steamed > boiled > raw trend (6.49–6.91), resulting from exposure of basic amino acid residues and ammonia production during protein denaturation, consistent with TVB-N trends [8,16,31].

TBARS values (Figure 1F) followed the order deep-fried (2.13 mg MDA/kg) > roasted (1.74 mg MDA/kg) > steamed (0.57 mg MDA/kg) > boiled (0.50 mg MDA/kg) > raw (0.33 mg MDA/kg). Deep-fried and roasted groups showed 548.0% and 431.7% increases, significantly higher than moist-heat groups (*p* < 0.05). The highest TBARS in the deep-fried group reflects both accelerated lipid oxidation at 170 °C and abundant substrate from absorbed frying oil (7.41 g/100 g fat) [13,16]. Moist-heat groups showed moderate TBARS increases due to lower temperatures (100 °C) and partial oxygen exclusion by steam/water [8,16]. Importantly, TBARS trends closely paralleled the crude fat content changes, suggesting that lipid content and oxidation degree jointly determine the formation of lipid oxidation-derived flavor compounds (aldehydes, ketones, alcohols) under different processing methods [3,8].

In summary, different thermal processing methods exerted distinct effects on *T. modestus* physicochemical properties through differential heat transfer mechanisms. Dry-heat processing (frying and roasting) caused more pronounced moisture loss, protein concentration, fat content changes (an especially dramatic increase in the deep-fried group), protein degradation, pH elevation, and lipid oxidation, while moist-heat processing (steaming and boiling) produced relatively moderate effects. These physicochemical differences provide the material basis for distinct flavor formation under each processing method—particularly, significant differences in fat content and TBARS indicate fundamental differences in substrate supply and reaction extent of lipid oxidation pathways, while protein degradation (TVB-N) and pH changes affect the generation of nitrogen-containing flavor compounds through Maillard reaction and Strecker degradation [3,8]. Mechanistically, these physicochemical gradients establish the precursor availability and reaction conditions that determine which flavor-forming pathways predominate under each processing method, as further elucidated in Section 3.5.8.

### 3.2. Effects of Different Processing Methods on Sensory Characteristics of T. modestus Muscle

**Figure 2:**
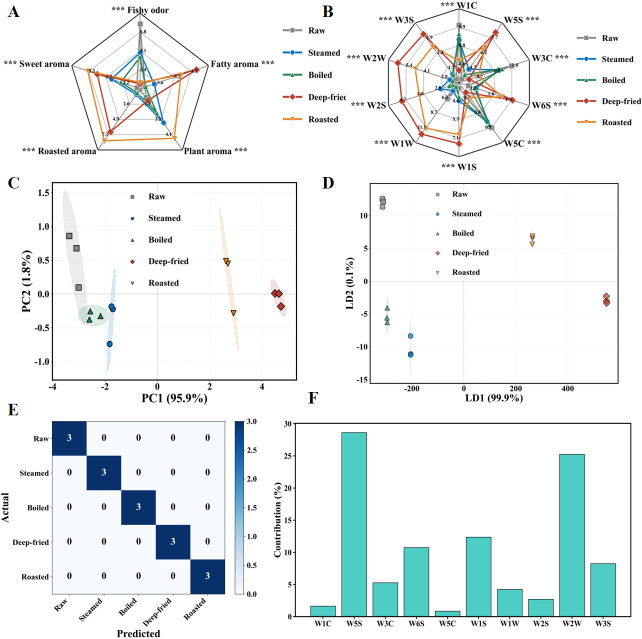
Sensory evaluation and electronic nose analysis of *Thamnaconus modestus* subjected to different processing methods. (**A**) QDA sensory evaluation radar chart; (**B**) electronic nose response signal radar chart; (**C**) principal component analysis (PCA) score plot of electronic nose; (**D**) linear discriminant analysis (LDA) plot of electronic nose; (**E**) classification accuracy confusion matrix; (**F**) sensor feature contribution.

A sensory evaluation radar chart for the five groups is shown in Figure 2A. QDA results indicated that different processing methods notably affected all five aroma attributes (fishy odor, fatty aroma, plant aroma, roasted aroma, sweet aroma) of *T. modestus* muscle (*p* < 0.05). Thermal processing markedly altered the monotonous fishy odor-dominated aroma profile of raw fish, enhancing fatty, roasted, and sweet aromas to varying degrees, with dry-heat processing (frying and roasting) producing the most dramatic changes.

For fishy odor, scores followed the order raw (7.60) > steamed (4.97) > boiled (4.56) > roasted (2.25) > deep-fried (2.13). The raw group exhibited the highest fishy odor score (7.60 ± 0.23), indicating high concentrations of TMA, dimethyl sulfide, and short-chain volatile fatty acids in fresh *T. modestus* muscle [11,12]. All thermally processed groups exhibited markedly reduced fishy odor (*p* < 0.05). Deep-fried and roasted groups showed the most pronounced reduction, attributable to effective thermal decomposition and volatilization of odorous compounds (TMA, sulfur-containing compounds) at high temperatures (170–200 °C) [8,13], combined with masking effects from abundant aromatic compounds (pyrazines, furans, aldehydes) generated via Maillard reaction and lipid oxidation [9,10]. The boiled group scored lower than the steamed group, possibly due to diffusion of water-soluble odorous compounds into the cooking liquid [16,17]. From a mechanistic perspective, the gradient reduction of fishy odor across processing methods directly reflects the progressive thermal decomposition of sulfur-containing precursors and TMA, whose volatilization rates increase nonlinearly with temperature, explaining why dry-heat methods (170–200 °C) were far more effective than moist-heat methods (100 °C) at eliminating off-odors. Chen et al. [10] reported similar trends in tilapia, where frying and roasting more effectively reduced fishy odor than steaming and boiling.

For fatty aroma, the deep-fried group scored highest (7.20 ± 0.60), considerably exceeding other groups (*p* < 0.05), followed by roasted (5.78), while raw (2.49) and boiled (2.54) scored lowest. The prominent fatty aroma in the deep-fried group resulted from extensive oil absorption (fat content increased from 1.16 to 7.41 g/100 g), with abundant lipid substrates undergoing intense oxidative degradation at 170 °C to generate aldehydes (hexanal, nonanal, (E)-2-nonenal), ketones (2-heptanone), and alcohols (1-octen-3-ol) [8,9,13]. This sensory observation was highly consistent with the TBARS trends—higher lipid oxidation degree correlates with stronger fatty aroma intensity.

For plant aroma, the roasted group scored highest (4.33), followed by steamed (3.40) and boiled (2.84), while raw (1.89) and deep-fried (2.07) scored lowest. Plant aroma primarily derives from short-chain aldehydes (hexanal, (E)-2-hexenal) produced through PUFA oxidative degradation, imparting grassy and green leaf notes [9,10]. Lower plant aroma in the deep-fried group may result from further oxidative decomposition of hexanal and masking by intense oily aroma [13,18].

Roasted aroma was the most distinguishing sensory attribute. The roasted group scored highest (8.04 ± 0.49), followed by deep-fried (6.70), both notably exceeding moist-heat groups and the raw group (*p* < 0.05). The raw group scored lowest (0.49), near the detection limit. Roasted aroma formation primarily results from Maillard reaction and caramelization, which accelerate markedly under high-temperature dry-heat conditions [8,14]. During 200 °C roasting, free amino acids (leucine, isoleucine, alanine) react with reducing sugars via Maillard reaction to generate pyrazines (2-methylpyrazine, 2,5-dimethylpyrazine), imparting characteristic roasted and nutty notes [8,9,14]. Strecker degradation-derived branched aldehydes (3-methylbutanal, 2-methylbutanal) also contribute to roasted aroma [8]. Steamed and boiled groups scored extremely low, as moist-heat temperature (100 °C) is far below the optimal ranges for Maillard reaction and caramelization (typically >120 °C) [10,14]. This temperature-dependent threshold effect mechanistically explains the sharp sensory divide between moist-heat and dry-heat products: only when processing temperatures exceed the activation energy barrier for α-amino ketone condensation and Amadori rearrangement can sufficient pyrazines and furans accumulate to produce perceptible roasted notes.

For sweet aroma, the roasted group scored highest (5.36), followed by deep-fried (4.49) and steamed (3.21), with raw lowest (0.91). The formation of sweet aroma is primarily attributed to Maillard reaction intermediates (furanones, lactones) and caramelization products (maltol, furfural) [8,14]. Under roasting (200 °C) and frying (170 °C) conditions, furans (2-pentylfuran, 2-acetylfuran) and furfural intermediates impart sweet and caramel notes [9,10].

In summary, sensory evaluation clearly revealed differential effects of processing methods on *T. modestus* aroma characteristics. The raw group was dominated by strong fishy odor with weak other attributes. Moist-heat processing moderately reduced fishy odor and enhanced plant aroma and sweet aroma. Dry-heat processing substantially weakened fishy odor while notably enhancing fatty aroma, roasted aroma, and sweet aroma. The roasted group exhibited the most balanced flavor profile with prominent roasted aroma, sweet aroma, and plant aroma; the deep-fried group was characterized by intense fatty aroma and elevated roasted aroma. These results indicate that lipid oxidation degree and Maillard reaction intensity are the key chemical factors determining flavor differences among processing methods [3,8]. Specifically, the observed sensory transformation from fishy-dominated to fatty/roasted-dominated profiles can be mechanistically attributed to two concurrent processes: (1) thermal volatilization and degradation progressively eliminating sulfur-based off-odor compounds, and (2) temperature-dependent activation of lipid oxidation, Strecker degradation, and Maillard reaction pathways generating pleasant aroma compounds that both mask residual off-odors and create desirable flavor notes. These sensory-derived flavor patterns were subsequently corroborated by the E-nose sensor response fingerprints (Section 3.3) and E-tongue taste profiles (Section 3.4), establishing a multi-dimensional sensory–instrumental consensus prior to molecular-level investigation by GC-IMS and GC-MS.

### 3.3. Effects of Different Processing Methods on E-Nose Fingerprint Characteristics

The E-nose sensor response radar chart for the five groups is shown in Figure 2B. The 10 MOS sensors (W1C, W5S, W3C, W6S, W5C, W1S, W1W, W2S, W2W, W3S) exhibited markedly different response values (R/R_0_) among processing methods (*p* < 0.05), indicating significant effects of thermal processing on volatile compound composition and concentration. Deep-fried and roasted groups generally exhibited considerably higher sensor responses than raw and moist-heat groups, reflecting abundant volatile flavor compound generation from intense lipid oxidation and Maillard reaction [8,9]. The raw group fingerprint was characterized by relatively prominent W1W (sulfur compounds) sensor response, corresponding to its fishy odor-dominated sensory characteristics.

For the W1W sensor (sulfur compounds), responses followed deep-fried (12.09) > roasted (11.15) > raw (6.43) > steamed (5.17) > boiled (4.50). Within moist-heat groups, W1W responses showed raw (6.43) > steamed (5.17) > boiled (4.50), consistent with fishy odor reduction trends in sensory evaluation. The raw group’s elevated W1W response primarily derives from high TMA and dimethyl sulfide concentrations [11,12]; steaming and boiling partially removed these low-boiling-point sulfur compounds through thermal evaporation and aqueous diffusion [16,17]. Despite higher W1W responses in the deep-fried (12.09) and roasted (11.15) groups, this did not indicate stronger fishy odor—under dry-heat conditions, high W1W responses mainly result from sulfur-containing heterocyclic compounds (thiazoles, thiophenes) generated via Maillard reaction and lipid thermal oxidation, which impart pleasant roasted and nutty notes rather than fishy odor [8,14].

For sensors reflecting lipid oxidation and Maillard reaction products (W5S, W1S, W2W, W2S), similar trends were observed. Taking W5S as an example, responses followed deep-fried (8.26) > roasted (6.79) > steamed (4.58) > boiled (4.01) > raw (3.63). Considerably higher responses in deep-fried and roasted groups correspond to enhanced fatty aroma and roasted aroma in sensory evaluation. These E-nose data were highly consistent with the TBARS trends—higher lipid oxidation degree correlates with higher corresponding sensor responses [3,8].

The PCA score plot (Figure 2C) showed that the first two principal components explained a cumulative variance of 97.7% (PC1 = 95.9%, PC2 = 1.8%), far exceeding the generally accepted threshold of 70% for adequate data representation. The five groups were clearly separated into three regions—raw, moist-heat groups (steamed and boiled), and dry-heat groups (deep-fried and roasted)—with non-overlapping 95% confidence ellipses, demonstrating effective discrimination among sample groups. PC1 loading analysis revealed that W1W, W5S, W1S, and W2W sensors (sensitive to sulfur compounds, nitrogen oxides, and alcohols) contributed most, confirming that lipid oxidation and Maillard reaction are key factors causing aroma differences [8,9].

LDA discrimination plot (Figure 2D) showed that the first two discriminant functions (LD1 and LD2) explained 99.9% and 0.1% of between-group variance, respectively. Five groups achieved complete separation with tight within-group clustering. Leave-one-out cross-validation (LOOCV) yielded an overall classification accuracy of 93.3% (14/15 samples correctly classified), confirming the model’s robustness and lack of overfitting, thereby validating the E-nose’s ability to reliably discriminate samples based on processing method [21,35]. Confusion matrix analysis (Figure 2E) demonstrated 100% classification accuracy with no misclassification. LDA feature contribution analysis (Figure 2F) showed W1W and W5S sensors contributed most, indicating that sulfur compound and lipid oxidation product concentrations are the most critical chemical basis for discriminating different processing methods. These E-nose discrimination patterns are further corroborated by the GC-MS volatile profiling (Section 3.5), where the aldehydes (hexanal, nonanal), furans, and pyrazines detectable by the W5S, W1S, and W2W sensors were found in greatest abundance in the deep-fried and roasted groups, providing molecular-level evidence for the sensor response differences observed here.

### 3.4. Effects of Different Processing Methods on E-Tongue Taste Characteristics

**Figure 3:**
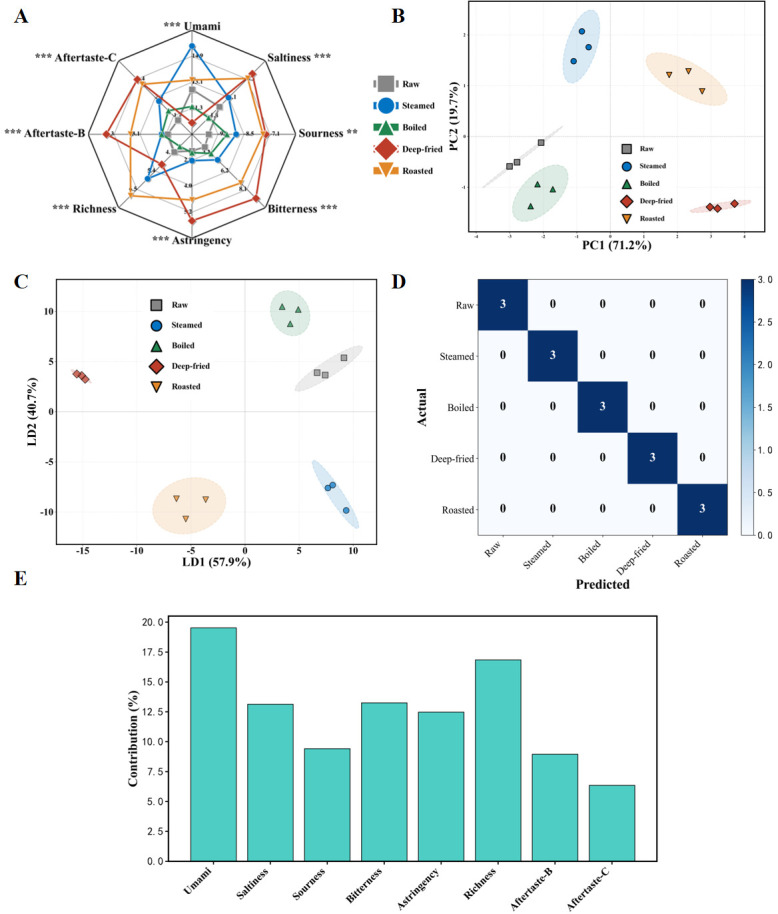
Electronic tongue analysis of *Thamnaconus modestus* subjected to different processing methods. (**A**) Electronic tongue response signal radar chart; (**B**) principal component analysis (PCA) score plot of electronic tongue; (**C**) linear discriminant analysis (LDA) plot of electronic tongue; (**D**) classification accuracy confusion matrix; (**E**) sensor feature contribution bar chart.

E-tongue analysis using the SA402B taste sensing system complemented the volatile flavor assessment by providing taste dimension data (Figure 3A). For umami response, the steamed group showed the highest value (15.66 ± 0.46), substantially exceeding other groups (*p* < 0.05), related to mild protein denaturation and release of free amino acids (glutamic acid, aspartic acid) and nucleotides (IMP) under 100 °C moist-heat conditions [33]. The boiled group showed appreciably reduced umami (11.45 ± 0.58) due to extensive leaching of water-soluble taste compounds into cooking liquid [16]. For richness (umami aftertaste), the roasted group showed the highest value (6.94 ± 0.31), possibly related to kokumi-enhancing compounds from Maillard reaction at 200 °C [8,14].

For bitterness, the deep-fried group showed the highest value (8.98 ± 0.51). Deep Maillard reaction at 170 °C produces bitter peptides and heterocyclic compounds, while high-temperature oil oxidation generates bitter aldehydes and ketones [8,13]. Correspondingly, the deep-fried group showed the highest astringency, bitterness aftertaste, and astringency aftertaste, consistent with its highest TBARS value (2.13 mg MDA/kg). The distinct bitterness and astringency profiles detected by the E-tongue in the deep-fried group (Figure 3A) are likely a direct consequence of the intense Maillard reaction and lipid oxidation products subsequently identified by GC-MS, including heterocyclic compounds (pyrazines, furans) and lipid-derived aldehydes such as (E,E)-2,4-decadienal and nonanal, and correlate with the high crude fat content (7.41 g/100 g) resulting from oil absorption [8,13]. For saltiness, deep-fried and roasted groups showed higher values due to concentration effects from substantial moisture loss.

The PCA score plot (Figure 3B) showed effective separation of the five groups in two-dimensional space. LDA classification accuracy reached 100.0% (Figure 3C), and the confusion matrix (Figure 3D) confirmed zero misclassification, with LOOCV accuracy of 86.7% (13/15 samples correctly classified), confirming the model’s generalizability and validating the E-tongue’s discriminative capacity for processing-dependent taste differences [33,34]. LDA feature contribution analysis (Figure 3E) indicated that umami and richness contributed most, followed by bitterness and astringency, suggesting that the umami–bitterness dynamics are the key dimensions distinguishing taste characteristics among processing methods.

### 3.5. Volatile Compound Profiles and Key Aroma-Active Compounds

To comprehensively characterize the volatile compound profiles of *T. modestus* under different thermal processing methods, two complementary gas-phase analytical platforms were employed: GC-IMS for rapid qualitative fingerprinting and visualization of volatile distribution patterns, and HS-SPME-GC-MS for detailed qualitative and quantitative identification of individual compounds. The results from both techniques are presented below in a unified analytical framework, with cross-platform corroboration discussed where applicable.

#### 3.5.1. GC-IMS Spectral Fingerprint Analysis

**Figure 4:**
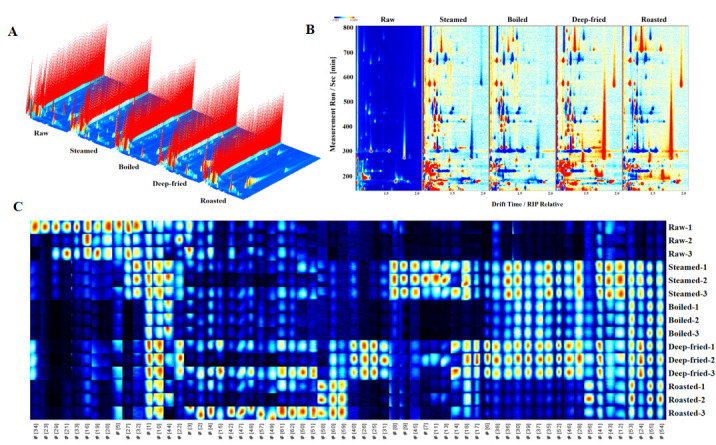
GC-IMS volatile compound fingerprint analysis of *Thamnaconus modestus* subjected to different processing methods. (**A**) GC-IMS three-dimensional spectrogram; (**B**) GC-IMS differential comparison plot; (**C**) GC-IMS Gallery Plot fingerprint.

Building upon the sensory and electronic sensing characterizations above, Table S1 and Figure 4 present GC-IMS results for *T. modestus* samples under different processing methods. Through combined retention index (RI) and drift time matching, 62 volatile compounds were identified, encompassing 10 categories including sulfur-containing compounds (5), aldehydes (16), alcohols (12), esters (7), ketones (8), furans (4), pyrazines (3), acids (3), amines (3), and diketones (1), with concentrations ranging from 7.15 to 5626.98 μg/kg (Table S1).

GC-IMS three-dimensional spectra (Figure 4A) showed raw group signals mainly concentrated in low-retention-time regions (Rt < 300 s), with relatively few peaks exhibiting uniform intensity. Steamed and boiled groups showed new peaks in moderate-retention-time regions (Rt 200–500 s) with enhanced overall intensity, attributable to moist-heat-promoted PUFA oxidative degradation and protein thermal denaturation. The deep-fried group exhibited numerous high-intensity peaks in high-retention-time regions (Rt > 400 s), with total volatile concentration reaching 41,590.94 μg/kg (1.7-fold of raw group’s 24,958.45 μg/kg), mainly resulting from synergistic Maillard reaction and intense lipid oxidation at high temperature. The roasted group similarly showed abundant peak distribution (37,351.93 μg/kg total concentration), with different peak patterns reflecting differential regulatory effects of dry-heat versus oil-heat processing on volatile compound formation pathways [8,14].

The two-dimensional topographic views (Figure 4B) further illustrated that raw group signals were mainly distributed in the region of drift time 1.0–1.5 with low overall intensity. Steamed and boiled groups showed enhanced signals in the Dt 1.0–1.5 region with new signal areas in the Dt 1.3–1.6 range. The deep-fried group exhibited large high-intensity signal areas in the Dt 1.2–1.8 interval (red/yellow regions), corresponding to substantial accumulation of Maillard reaction intermediates and deep lipid oxidation products. The roasted group showed significant signal enhancement in the Dt > 1.3 region with spatial distribution differences from the deep-fried group.

#### 3.5.2. GC-IMS Gallery Plot Fingerprint Analysis

The Gallery Plot fingerprint (Figure 4C) presented 62 volatile compounds across 15 samples (5 groups × 3 replicates) in matrix format, with brightness indicating signal intensity (bright = high concentration, dark = low concentration). Within-group replicates showed highly consistent fingerprint patterns, demonstrating good analytical reproducibility. Aldehydes (16 compounds) were the most detected category, primarily derived from PUFA oxidative degradation [8,9]. Total aldehyde concentrations followed deep-fried (16,021.34 μg/kg) > roasted (12,971.23 μg/kg) > boiled (6539.27 μg/kg) > steamed (4804.03 μg/kg) > raw (4166.55 μg/kg). Deep-fried group aldehyde concentration was approximately 3.8-fold that of the raw group, reflecting the mechanistic consequence of dramatically elevated lipid substrate availability (7.41 g/100 g fat from oil absorption) coupled with intense radical-mediated PUFA β-scission at 170 °C. Representative aldehydes showed dramatic processing-dependent increases: heptanal (36.7-fold in deep-fried), nonanal (6.8-fold in roasted), and benzaldehyde (9.5-fold in deep-fried), the latter possibly related to phenylalanine Strecker degradation. Detailed compound concentrations are provided in Table S1.

Other notable trends included the following: 1-octen-3-ol increased 6.6-fold in the roasted group, confirming enhanced linoleic acid oxidative cleavage at high temperatures [9,10]; dimethyl sulfide decreased in all thermally processed groups; and pyrazines (characteristic Maillard reaction markers) were detected predominantly in dry-heat groups (Table S1), imparting nutty and roasted notes [8,14].

Total volatile concentrations followed deep-fried (41,590.94 μg/kg) > roasted (37,351.93 μg/kg) > boiled (32,235.80 μg/kg) > steamed (26,065.91 μg/kg) > raw (24,958.45 μg/kg). Higher concentrations in dry-heat groups relate to synergistic acceleration of Maillard reaction, lipid oxidation, protein thermal degradation, and Strecker degradation [8,9,14].

The above GC-IMS fingerprint analysis provided a rapid overview of volatile distribution patterns and semi-quantitative trends. To further validate these observations and achieve precise compound identification and quantification, HS-SPME-GC-MS analysis was performed as detailed below. Notably, the two platforms showed excellent agreement in identifying major volatile categories (aldehydes, alcohols, ketones) and their processing-dependent trends, thereby strengthening the reliability of the overall volatile profiling.

#### 3.5.3. HS-SPME-GC-MS Analysis and Hierarchical Clustering

**Figure 5:**
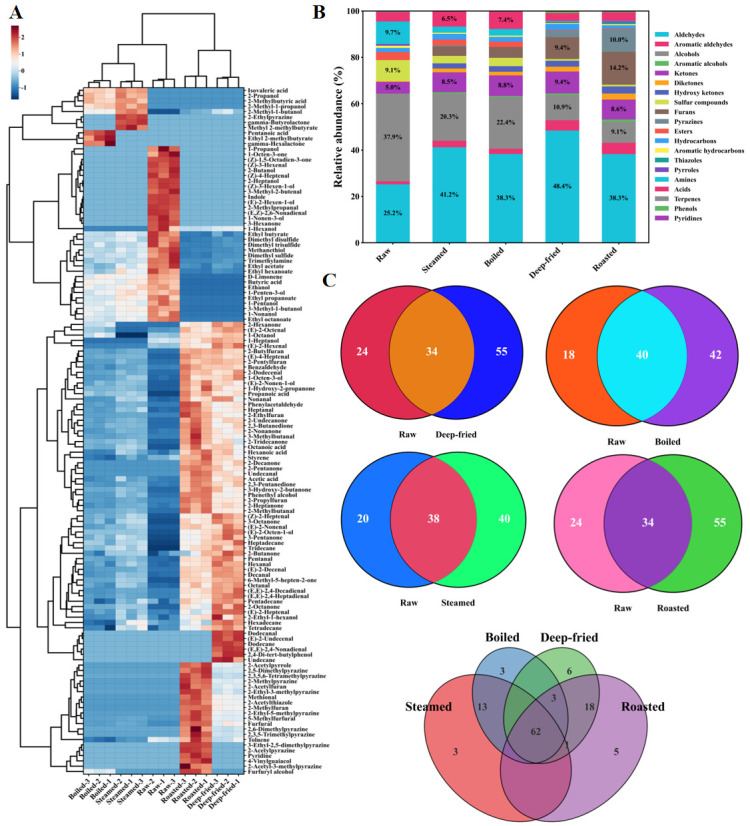
GC-MS volatile compound analysis of *Thamnaconus modestus* subjected to different processing methods. (**A**) Hierarchical clustering heatmap analysis; (**B**) stacked bar chart of volatile compound category percentages; (**C**) Venn diagram of volatile compounds among different processing groups.

As shown in Table S2 and Figure 5A, HS-SPME-GC-MS identified 129 volatile compounds across 20 chemical categories (*p* < 0.05). The number of detected compounds differed significantly among groups: raw (58), steamed (78), boiled (82), deep-fried (89), and roasted (89). Dry-heat groups showed considerably more compound types than raw and moist-heat groups, indicating that high-temperature dry-heat conditions activated additional chemical reaction pathways (Maillard reaction, Strecker degradation) promoting novel volatile compound generation [8,9]. Total volatile contents followed roasted (5465.05 μg/kg) > deep-fried (4983.52 μg/kg) > steamed (2226.53 μg/kg) > boiled (1767.22 μg/kg) > raw (1675.05 μg/kg). Roasted group content was approximately 3.3-fold that of raw, confirming that thermal processing, especially high-temperature dry heat, substantially promotes volatile flavor compound generation.

The hierarchical clustering heatmap using Ward’s method and Euclidean distance clearly divided the five groups into three branches: the raw group formed a separate branch characterized by sulfur-containing compounds (dimethyl sulfide, dimethyl disulfide, dimethyl trisulfide), amines (trimethylamine), and short-chain alcohols (1-pentanol, 1-hexanol), imparting fishy off-odor [11,12]. Steamed and boiled groups clustered together, reflecting similar modification effects of moist-heat processing (100 °C) with moderate sulfur compound reduction and aldehyde increase. Deep-fried and roasted groups clustered in another branch, showing high abundance (orange-red to dark-red) in aldehyde, furan, and pyrazine regions, indicating effective promotion of PUFA deep oxidative degradation and non-enzymatic browning reactions at 170–200 °C [8,14]. These clustering patterns were highly consistent with the sensory evaluation and E-nose PCA grouping patterns, providing molecular-level chemical explanations for observed flavor differences [10,16]. These GC-MS results showed excellent concordance with the GC-IMS fingerprint analysis (Section 3.5.1 and Section 3.5.2): both platforms consistently identified aldehydes as the dominant volatile category, confirmed the highest total volatile concentrations in dry-heat groups, and revealed similar processing-dependent trends for key compounds including hexanal, nonanal, and 1-octen-3-ol.

#### 3.5.4. Category Composition and Content Distribution Characteristics

Figure 5B presents the relative abundance distribution of volatile compound categories as stacked bar charts. Individual compound concentrations are provided in Table S2 (Supplementary Materials). The 129 identified compounds were classified into 20 categories including aldehydes, aromatic aldehydes, alcohols, aromatic alcohols, ketones, diketones, hydroxy ketones, sulfur-containing compounds, furans, pyrazines, esters, hydrocarbons, aromatic hydrocarbons, thiazoles, pyrroles, amines, acids, terpenes, phenols, and pyridines. Top three categories by relative abundance: raw group—alcohols (37.9%), aldehydes (25.2%), amines (9.7%); steamed group—aldehydes (41.2%), alcohols (20.3%), ketones (8.5%); boiled group—aldehydes (38.3%), alcohols (22.4%), ketones (8.8%); deep-fried group—aldehydes (48.4%), alcohols (10.9%), ketones (9.4%); roasted group—aldehydes (38.3%), furans (14.2%), pyrazines (10.0%).

Aldehydes were the dominant category across all groups, with relative abundance increasing from 25.2% in raw to 48.4% in deep-fried, attributable to thermal oxidative degradation of PUFAs (linoleic acid, arachidonic acid, EPA, DHA) generating characteristic lipid oxidation products (hexanal, nonanal, (E)-2-nonenal, (E,E)-2,4-decadienal) [8,14]. Pyrazines were barely detected in raw, steamed, and boiled groups but contributed significantly in deep-fried and roasted groups—compounds like 2-methylpyrazine, 2,5-dimethylpyrazine, and 2,3,5-trimethylpyrazine are characteristic Maillard reaction and Strecker degradation products imparting nutty and roasted notes [9,18]. Furans similarly showed dry-heat groups >> moist-heat groups and raw, with the roasted group showing the highest furan proportion, correlating with deep Maillard reaction and caramelization activation at 200 °C [40]. The exclusive detection of pyrazines and abundance of furans in dry-heat groups can be mechanistically explained by the temperature-dependent kinetics of these pathways: pyrazine formation via α-amino ketone self-condensation requires temperatures above 120–140 °C for significant accumulation, while furan generation from Amadori product 1,2-enolization is similarly favored at elevated temperatures where sugar fragmentation rates become kinetically competitive [8,41]. Conversely, sulfur-containing compounds showed dramatic decline from significant levels in raw to near-lowest levels in deep-fried and roasted groups, attributable to thermal denaturation disrupting protein cross-linked structures and releasing low-molecular-weight sulfur compounds to gas phase [13], corresponding to significant fishy odor reduction in sensory evaluation.

#### 3.5.5. Venn Diagram Analysis of Shared and Unique Volatile Compounds

Venn diagram analysis (Figure 5C) revealed shared and unique volatile compounds among the five groups. Twenty-nine compounds were shared across all groups (22.5% of total), mainly including hexanal, nonanal, 1-octen-3-ol, and dimethyl sulfide, constituting the basic volatile matrix of *T. modestus* muscle. Unique compound numbers: raw (15), steamed (3), boiled (3), deep-fried (6), and roasted (5). Dry-heat groups possessed the most unique compounds. The roasted group’s five unique compounds included 2-acetylpyrazine (roasted, popcorn-like), 2-acetyl-3-methylpyrazine (nutty), 4-vinylguaiacol (smoky, clove-like), and 3-ethyl-2,5-dimethylpyrazine (nutty, roasted)—characteristic Maillard reaction and caramelization products generated only at sufficiently high temperatures [18,40]. The deep-fried group’s six unique compounds mainly included 2,4-di-tert-butylphenol, (E)-2-undecenal, and dodecanal—the former is a characteristic oil thermal oxidation marker, while the latter two are deep oxidative degradation products of long-chain fatty acids at 170 °C [42]. The raw group’s unique compounds—(Z)-4-heptenal and 1-octen-3-one—are typical fresh fish volatile markers; the former derives from lipoxygenase-catalyzed ω-3 PUFA oxidation with strong fishy odor characteristics, while the latter originates from arachidonic acid enzymatic oxidation [39,43]. Both compounds disappeared in all thermally processed groups due to thermal instability, explaining mechanistically how thermal processing effectively reduces fishy odor.

#### 3.5.6. OPLS-DA-Based Screening of Characteristic Volatile Markers

**Figure 6:**
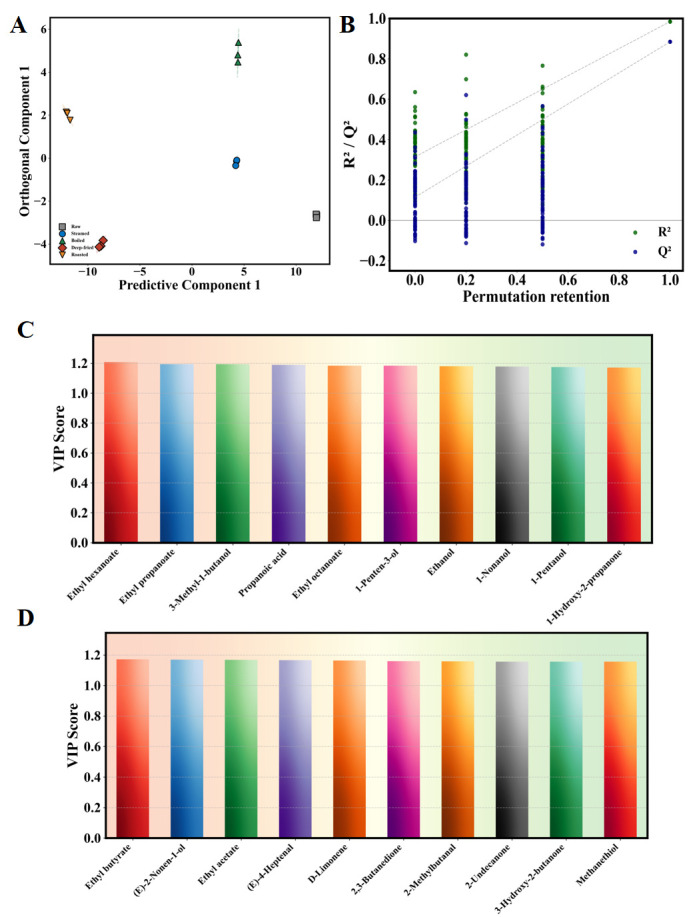
Multivariate statistical analysis based on GC-MS of *Thamnaconus modestus* subjected to different processing methods. (**A**) OPLS-DA score plot; (**B**) OPLS-DA permutation test plot; (**C**) top 1–10 characteristic volatile compounds based on VIP value > 1; (**D**) top 11–20 characteristic volatile compounds based on VIP value > 1.

An OPLS-DA supervised multivariate statistical model was constructed based on full-spectrum volatile data to evaluate compound contributions to classification and screen characteristic markers [27,28]. The OPLS-DA score plot (Figure 6A) showed clear separation of five groups with tight within-group clustering and non-overlapping 95% confidence ellipses. The raw group was located in the rightmost region with maximum discriminant distance from all thermally processed groups; steamed and boiled groups were separated in the orthogonal component direction but close in the predictive component direction; deep-fried and roasted groups were located in the upper-left and lower-left regions, respectively. The OPLS-DA model exhibited excellent goodness of fit and predictive ability, with R^2^X = 0.891, R^2^Y = 0.997, and Q^2^ = 0.953, indicating that the model explained 99.7% of the between-group variation with 95.3% predictive accuracy under seven-fold cross-validation. A permutation test (n = 200; Figure 6B) confirmed model validity—all permuted R^2^ and Q^2^ values were substantially lower than the original model values, with R^2^ intercept = 0.218 and Q^2^ intercept = −0.341, both well below the recommended thresholds (R^2^ intercept < 0.4, Q^2^ intercept < 0.05), providing strong evidence against overfitting [28]. Based on the VIP > 1 criterion, 78 characteristic differential volatile compounds were screened, with the top 10 markers shown in Figure 6C and the remaining markers in Figure 6D. Top five VIP compounds: Ethyl hexanoate (VIP = 1.208), Ethyl propanoate (VIP = 1.194), 3-Methyl-1-butanol (VIP = 1.193), Propanoic acid (VIP = 1.189), Ethyl octanoate (VIP = 1.184). These high-VIP compounds encompassed lipid oxidation products, sulfur-containing compounds, esters, and terpenes, reflecting systematic flavor profile remodeling through multiple metabolic pathways [27,28].

#### 3.5.7. OAV-Based Identification of Key Aroma Compounds

**Figure 7:**
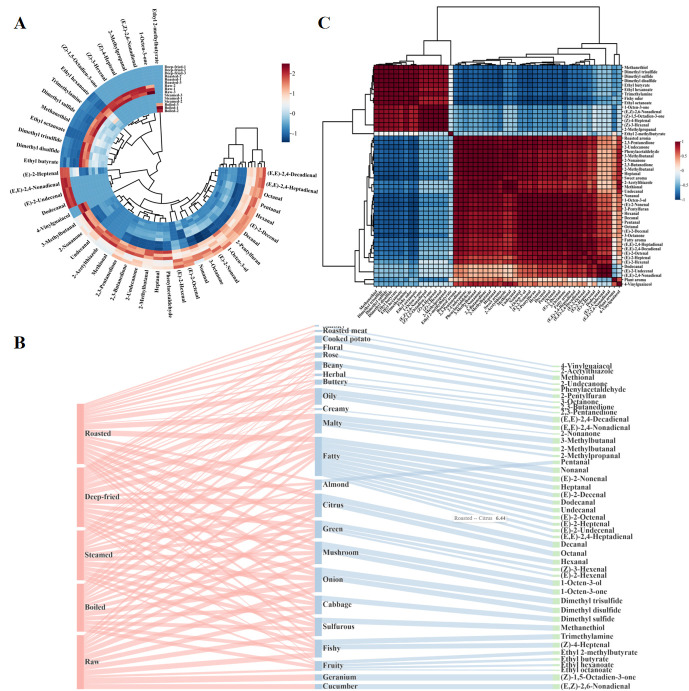
Key aroma compounds analysis of *Thamnaconus modestus* subjected to different processing methods. (**A**) Hierarchical clustering heatmap of key aroma compounds based on OAVs; (**B**) Sankey diagram of key aroma compounds; (**C**) Pearson correlation heatmap between key aroma compounds and sensory attributes.

OAV (compound concentration/odor threshold) ≥ 1 indicates compounds exceeding the human olfactory perception threshold, identified as key aroma-active compounds [29,36,37]. The external standard method with six-gradient standard curves (R^2^ ≥ 0.995, Table S4) was employed for accurate quantification. From 129 volatile compounds, 45 key aroma compounds with OAV ≥ 1 were identified across multiple chemical categories (Figure 7A, Table S3).

In the raw group, the highest OAV compounds were mainly sulfur-containing compounds and amines: dimethyl trisulfide (OAV = 1063.3, onion-like, fishy), methanethiol (OAV = 378.0, sulfurous, putrid), dimethyl disulfide (OAV = 322.1, onion-like, fishy), and trimethylamine (OAV = 270.7, fishy, ammonia-like) [11,12]. These are core contributors to characteristic fishy off-odor in fresh *T. modestus*, explaining the significantly higher fishy odor score (7.60) in the raw group. The raw group also showed high OAV for (Z)-4-heptenal (OAV = 395.3, fishy, creamy) and 1-octen-3-one (OAV = 610.0, mushroom-like, metallic), both primary products of arachidonic acid lipoxygenase-catalyzed oxidation and characteristic fresh marine fish flavor markers [43].

After thermal processing, sulfur compounds and TMA showed pronounced gradient decline following raw >> steamed ≈ boiled >> deep-fried > roasted. This resulted from the synergistic effects of thermal denaturation disrupting protein cross-linked structures (releasing bound low-molecular-weight sulfur compounds) [13] and higher dry-heat temperatures (170–200 °C vs. 100 °C) accelerating thermal decomposition and Strecker degradation-mediated transformation [14]. Conversely, lipid oxidation-derived key aroma compounds—hexanal (grassy, fatty), nonanal (fatty, citrus), (E)-2-nonenal (fatty, cucumber), (E,E)-2,4-decadienal (oily, fishy), and 1-octen-3-ol (mushroom-like, earthy)—showed markedly elevated OAV in all thermally processed groups. (E,E)-2,4-decadienal typically forms from linoleic acid radical-mediated oxidative degradation and is a core contributor to characteristic oily notes in fried foods [39]. 1-Octen-3-ol mainly derives from arachidonic acid oxidative degradation, accelerated by heating [43]. Roasted and deep-fried groups additionally showed diketones—2,3-butanedione (buttery, creamy) and 2,3-pentanedione (buttery, caramel)—as key aroma contributors (OAV > 1), imparting unique buttery and caramel notes. These diketones typically form from further degradation of Maillard reaction intermediates, related to Amadori rearrangement product cleavage [8,9,44]. The roasted group showed the highest OAV for 3-methylbutanal (malty, chocolate-like), a characteristic Strecker aldehyde from leucine degradation, indicating 200 °C roasting most effectively activated this pathway [45].

In summary, different processing methods fundamentally reshaped the *T. modestus* aroma profile through differential effects on key aroma compound generation: the raw group was dominated by fishy and sulfurous notes; moist-heat groups were characterized by grassy and fatty notes; dry-heat groups featured fatty, roasted, and nutty notes. This aroma transformation pattern was highly consistent with the sensory evaluation and E-nose fingerprint analysis results. The OAV data thus provide compound-level mechanistic evidence that the observed sensory changes are driven by the differential activation of three key metabolic pathways—lipid oxidative degradation, amino acid Strecker degradation, and Maillard reaction—each with distinct temperature thresholds and substrate requirements, as systematically elaborated in Section 3.5.8. It should be noted that these OAVs were calculated using odor thresholds determined in water [36,37], which may not fully represent the complexity of perception in the fish muscle matrix, potentially over- or underestimating the actual perceptual contribution of individual compounds; (2) potential synergistic and antagonistic interactions between co-existing volatile compounds were not accounted for in the additive OAV model; and (3) individual variability in olfactory sensitivity and the influence of the food matrix on compound release kinetics may further affect the accuracy of OAV-based predictions. These caveats should be considered when interpreting the OAV-derived key aroma compound rankings.

#### 3.5.8. Metabolic Formation Pathways and Flavor Formation Mechanisms

Based on identified key aroma compounds and the literature [8,14,39,40,41,43,45,46], a Sankey diagram (Figure 7B) was constructed to provide a unified mechanistic framework that integrates the physicochemical, sensory, E-nose, GC-IMS, and GC-MS findings presented above by visualizing the potential metabolic formation pathways, with flow line width proportional to OAVs, illustrating three-layer associations among processing methods (left), aroma descriptors (middle), and key aroma compounds (right). The raw group’s major aroma flows concentrated on ‘fishy’ and ‘sulfurous’ categories; as processing shifted from moist heat to dry heat, aroma flows systematically migrated from ‘fishy/sulfurous’ toward ‘grassy/fatty’ then toward ‘nutty/caramel/roasted’. Specifically, the Sankey diagram illustrates how the choice of thermal processing method dictates the dominant flavor pathway: moist-heat methods (steaming, boiling) primarily channel precursors through the lipid oxidation pathway, resulting in the accumulation of grassy/fatty aldehydes (hexanal, nonanal, (E)-2-nonenal), whereas the high temperatures of dry-heat methods (deep-frying, roasting) provide the activation energy to simultaneously engage the Maillard reaction and Strecker degradation pathways, leading to the formation of roasted/nutty pyrazines (2-methylpyrazine, 2,5-dimethylpyrazine) and furans (2-pentylfuran, furfural) [8,14,41]. Lipid oxidative degradation pathway was the primary source of aldehydes and some alcohols. *T. modestus* muscle is rich in PUFAs (linoleic acid, arachidonic acid, EPA, DHA) [2,3]. During thermal processing, these PUFAs undergo free radical chain reactions to form lipid hydroperoxides, which further cleave via β-scission to generate short-chain volatile aldehydes, alcohols, and ketones [39]. Specifically, linoleic acid thermal oxidation mainly generates hexanal, (E)-2-nonenal, and (E,E)-2,4-decadienal; oleic acid oxidation generates nonanal and octanal; arachidonic acid oxidation generates 1-octen-3-ol and (Z)-4-heptenal [43].

The amino acid Strecker degradation pathway was the major source of branched aldehydes and some sulfur compounds. Under heating, α-dicarbonyl Maillard reaction intermediates (3-deoxyglucosone, methylglyoxal) react with free amino acids through oxidative decarboxylation to generate Strecker aldehydes with one fewer carbon [45]. Leucine generates 3-methylbutanal, phenylalanine generates phenylacetaldehyde, and methionine generates methanethiol and dimethyl sulfide [45,46]. The roasted group’s highest 3-methylbutanal OAV indicates 200 °C roasting most effectively activated Strecker degradation [45].

The Maillard reaction pathway was the main route for pyrazines, furans, and nitrogen-containing heterocyclic compounds. Maillard reaction, initiated by nucleophilic addition between reducing sugars and amino acids/proteins, proceeds through Schiff base formation, Amadori rearrangement, sugar fragmentation, amino acid degradation, and aldol condensation to generate various nitrogen-containing heterocyclic compounds and furans [8,41]. Pyrazines (2-methylpyrazine, 2,5-dimethylpyrazine, 2,3,5-trimethylpyrazine, 2,6-dimethylpyrazine, 2-ethyl-3-methylpyrazine) were abundantly detected only in deep-fried and roasted groups, generated through α-amino ketone self-condensation, imparting nutty and roasted notes [8]. Furans (furfural, 5-methylfurfural, 2-acetylfuran) mainly derive from sugar caramelization and Amadori product 1,2-enolization, imparting caramel and sweet notes to dry-heat samples [40,41].

The sulfur compound thermal volatilization and transformation pathway also contributed significantly. Fresh *T. modestus* muscle contains high concentrations of dimethyl sulfide, dimethyl disulfide, dimethyl trisulfide, and TMA, partially in free form and partially bound to proteins non-covalently [11,12]. Heating causes protein thermal denaturation and cross-linked structure disruption, releasing bound sulfur compounds that evaporate to gas phase, reducing residual concentrations and OAVs [13]. Additionally, accelerated methionine Strecker degradation at high temperature further transforms sulfur compounds. These synergistic pathways caused gradient OAV decline (raw >> steamed ≈ boiled >> deep-fried > roasted), mechanistically explaining how thermal processing improves fishy odor. In summary, different processing methods formed distinct volatile composition profiles and aroma characteristics through differential regulation of lipid oxidative degradation, amino acid Strecker degradation, Maillard reaction, caramelization, and sulfur compound volatilization. Moist-heat processing (100 °C) mainly modified flavor through promoting lipid oxidation and accelerating sulfur compound volatilization, producing mild processed flavors dominated by grassy and fatty notes. This mechanistic selectivity arises because 100 °C provides sufficient thermal energy for homolytic cleavage of PUFA peroxyl radicals (activation energy ~80–120 kJ/mol) but is insufficient for the condensation reactions (>120 °C) required for pyrazine and furan formation. Dry-heat processing (170–200 °C) not only markedly accelerated lipid oxidation but also strongly activated Maillard reaction, Strecker degradation, and caramelization, generating abundant pyrazines, furans, and Strecker aldehydes, imparting rich roasted, nutty, and caramel characteristics.

#### 3.5.9. Pearson Correlation Analysis Between Key Aroma Compounds and Sensory Attributes

Pearson correlation analysis was performed between concentrations of the top 15 OAV-ranked key aroma compounds and five sensory attributes (fishy odor, fatty aroma, plant aroma, roasted aroma, sweet aroma) (Figure 7C). A correlation heatmap with clustering reordered variables, displaying correlation coefficients as colored spheres in the upper triangle (red = positive, blue = negative; larger sphere = stronger correlation) and numerical values in the lower triangle (* *p* < 0.05, *p* < 0.01). Sulfur compounds (dimethyl trisulfide, dimethyl disulfide, dimethyl sulfide, methanethiol) and trimethylamine concentrations showed strong positive correlations with fishy odor scores, confirming these compounds as the core molecular basis for *T. modestus* fishy off-odor from a chemical–sensory perspective. These compounds showed significant negative correlations with roasted aroma and sweet aroma, indicating antagonistic relationships—sulfur compound concentration decline directly accompanies roasted aroma enhancement [11,12,13].

Lipid oxidation-derived key aroma compounds (hexanal, nonanal, (E)-2-nonenal, (E,E)-2,4-decadienal, 1-octen-3-ol) showed significant positive correlations with fatty aroma and negative correlations with fishy odor. This indicates that heat-induced lipid oxidation played dual roles: generating abundant aldehydes and alcohols to enhance fatty notes while accompanying sulfur compound volatilization to weaken fishy perception—synergistically driving aroma transformation from ‘fishy-dominated’ to ‘fatty-dominated’ [39,43]. Maillard reaction and Strecker degradation products (3-methylbutanal, 2,3-butanedione, 2,3-pentanedione) showed significant positive correlations with roasted aroma and sweet aroma (*p* < 0.05) and strong negative correlations with fishy odor, revealing the key role of the Maillard reaction pathway in imparting roasted and sweet characteristics to dry-heat-processed fish products [8,9,45]. Clustering analysis divided all sensory attributes and key aroma compounds into two main branches: sulfur compounds and amines positively correlated with fishy odor clustered on one side; lipid oxidation products and Maillard reaction products positively correlated with roasted/sweet aroma clustered on the other, consistent with the OPLS-DA score plot showing significant raw versus dry-heat group separation [27,28].

The integration of sensory evaluation and OAV analysis further strengthened the interpretation of flavor differences among processing methods. Specifically, the roasted group’s highest roasted aroma score (8.04) was directly supported by its highest OAVs for 3-methylbutanal (Strecker aldehyde) and 2,3-butanedione (Maillard intermediate), while the deep-fried group’s dominant fatty aroma (7.20) corresponded to its highest OAV for (E,E)-2,4-decadienal and hexanal. The concordance between the sensory-perceived fishy odor gradient (raw >> moist-heat >> dry-heat) and the OAV-based sulfur compound gradient (dimethyl trisulfide OAV: raw 1063.3 >> steamed/boiled >> deep-fried/roasted) provided compound-level validation of the sensory panel’s assessments. This multi-dimensional correspondence between sensory attributes and OAV-ranked compounds confirmed that the integrated flavoromics framework yields internally consistent and scientifically robust characterization of processing-dependent flavor transformations.

In summary, Pearson correlation analysis revealed quantitative relationships between key aroma compound concentrations and sensory attribute variations from a chemical–sensory perspective, providing direct statistical evidence for the molecular mechanisms underlying thermal processing effects on *T. modestus* flavor, and identifying actionable target compounds for precise flavor quality control. From a mechanistic standpoint, the strong negative correlation between sulfur compounds and roasted/sweet aroma attributes confirmed that these two compound classes occupy opposing ends of the metabolic transformation spectrum, providing quantitative validation of the pathway-level analysis presented in Section 3.5.8.

## 4. Conclusions

Addressing the three research questions posed in the Introduction, this study systematically investigated the effects of four thermal processing methods (steaming, boiling, deep-frying, and roasting) on the volatile flavor profile of *T. modestus* using an integrated multi-instrumental flavoromics platform. The results confirmed the hypothesis that different thermal processing methods differentially alter volatile profiles through distinct pathway activation. Fresh *T. modestus* muscle exhibited pronounced fishy off-odor primarily attributed to high concentrations of sulfur-containing compounds and trimethylamine. Thermal processing effectively reduced fishy off-odor while enhancing pleasant aroma attributes, with dry-heat methods (deep-frying and roasting) showing the most pronounced sensory improvement. GC-IMS and HS-SPME-GC-MS identified 62 and 129 volatile compounds, respectively, from which OPLS-DA screened 78 characteristic differential markers (VIP > 1) and OAV analysis identified 45 key odorants (OAV ≥ 1). Sulfur-containing compound concentrations exhibited a gradient decline across processing methods (raw >> steamed ≈ boiled >> deep-fried > roasted), while lipid oxidation- and Maillard reaction-derived compounds showed corresponding increases.

Two distinct flavor modulation patterns were identified: moist-heat methods (steaming, boiling) produced grassy/fatty notes through moderate lipid oxidation, while dry-heat methods (deep-frying, roasting) generated characteristic roasted/nutty notes via synergistic activation of Maillard reaction, Strecker degradation, and caramelization at elevated temperatures (170–200 °C). Pearson correlation analysis confirmed the quantitative relationships between key aroma compound concentrations and sensory attribute variations, demonstrating that different processing methods achieved systematic flavor profile transformation from “fishy-dominated” to “fatty/roasted-dominated” through differential regulation of multiple metabolic pathways.

As the first comprehensive flavoromics-based investigation of volatile compounds in *T. modestus* under multiple thermal processing methods, these findings provide a scientific basis for diversifying *T. modestus* deep-processing beyond over-reliance on a single roasting method toward steaming, boiling, and frying products, and offer theoretical support for precise flavor quality control and process optimization. Practically, these findings suggest that (1) steaming preserves umami while mildly reducing fishiness, making it suitable for products targeting delicate flavor preferences; (2) deep-frying generates intense fatty aroma but at the cost of substantial oil absorption; and (3) roasting produces the most balanced flavor profile with the strongest desirable aroma attributes, supporting its continued use while also demonstrating that other thermal methods can create distinct, marketable flavor profiles. Future research integrating *lipidomics* and *proteomics* to elucidate flavor precursor transformation mechanisms, combined with consumer preference studies based on targeted key aroma compound regulation, will further advance the high-quality development of the *T. modestus* processing industry.

Several limitations of this study should be acknowledged. First, the use of three biological replicates (n = 3), while consistent with established volatile analysis protocols [10,16,20], may limit statistical power for detecting subtle differences; future studies should employ larger sample sizes to enhance robustness. Second, the OAV calculations relied on odor thresholds determined in water, which may not accurately represent compound perception in the fish matrix; matrix-matched thresholds would improve prediction accuracy. Third, this study did not include sensory–instrumental regression modeling (e.g., partial least squares regression), which would provide more rigorous quantification of compound–sensory relationships. Fourth, the absence of real-time process monitoring limited our ability to track dynamic changes in volatile compound formation during thermal processing.

Future research should address these limitations by (1) integrating lipidomics and proteomics to provide deeper mechanistic understanding of flavor precursor transformation during thermal processing; (2) conducting consumer preference studies based on targeted regulation of key aroma compounds to optimize product acceptability; (3) developing partial least squares regression models between instrumental measurements and sensory attributes; and (4) applying real-time volatile monitoring techniques (e.g., proton-transfer-reaction mass spectrometry) to elucidate the temporal dynamics of flavor formation under different processing conditions.

## Data Availability

The original contributions presented in the study are included in the article/Supplementary Materials, further inquiries can be directed to the corresponding authors.

## Supplementary Materials

The following supporting information can be downloaded at https://www.mdpi.com/article/10.3390/foods15081352/s1: Table S1. Volatile compounds identified by GC-IMS in *Thamnaconus modestus* subjected to different processing methods. Table S2. Volatile compounds identified by HS-SPME-GC-MS in *Thamnaconus modestus* subjected to different processing methods. Table S3. Key aroma compounds screened by odor activity value (OAV) in *Thamnaconus modestus* subjected to different processing methods. Table S4. Standard curve parameters for external standard quantification.
