# Supplementary material for: Comprehensive Characterization of Volatile Flavor Compounds in Thamnaconus modestus Under Different Thermal Processing Methods: A Multi-Instrumental Flavoromics Approach
Source: Foods. 2026 Apr 13;15(8):1352. doi: 10.3390/foods15081352 (PMC13114293; doi:10.3390/foods15081352)
Supplement: Supplementary file 1 [file foods-15-01352-s001.zip › foods-4181880-supplementary.pdf]

Comprehensive characterization of volatile flavor compounds in Thamnaconus modestus under different thermal processing methods: A multi-instrumental flavoromics approach

Qinmei Fang [a,*], LingKe [a], Li Bian [b], Hongshu Chi [a], Ximin Qiu [c], Yongcong Chen [a], Shuigen Li [c],

Siqing Chen [a], Shaohua Shi [b,*]

[a] Biotechnology Research Institute, Fujian Academy of Agricultural Sciences, Fuzhou, Fujian, 350011, China.

[b] State Key Laboratory of Mariculture Biobreeding and Sustainable Goods, Yellow Sea Fisheries Research Institute, Chinese Academy of Fishery Sciences, Qingdao, Shandong, 266071, China.

[c] Fujian Fisheries Technology Extension Center, Fuzhou,Fujian, 350011, China.

∗ **Correspondence:** Qinmei Fang, E-mail: faasfang@126.com.

∗ **Correspondence:** Shaohua Shi, E-mail: shaohua115@163.com.

[*] Corresponding author at: Biotechnology Research Institute, Fujian Academy of Agricultural Sciences, Fuzhou, Fujian, 350011, China.

E-mail address: faasfang@126.com (Qinmei Fang).

[*] Corresponding author at: Biotechnology Research Institute, Fujian Academy of Agricultural Sciences, Fuzhou, Fujian, 350011, China.

E-mail address: shaohua115@163.com (Shaohua Shi).

Table S1 Volatile compounds identified by GC-IMS in Thamnaconus modestus subjected to different processing methods.

No.	Compound	CAS No.	Category	Molecular formula	MW	Rt (sec)	RI	Dt (RIPrel)	Identification	Odor description	Raw (μg/kg)	Steamed (μg/kg)	Boiled (μg/kg)	Deep-fried (μg/kg)	Roasted (μg/kg)
1	Methanethiol	74-93-1	Sulfur compounds	CH4S	48.1	99.077	116.5	10.673	RI + Drift time	Putrid, rotten egg	771.22±60.91	716.72±22.68	860.74±3.22	852.87±21.78	1054.41±34.68
2	Dimethyl sulfide	75-18-3	Sulfur compounds	C2H6S	62.1	124.035	215.4	13.924	RI + Drift time	Cabbage, sulfurous	349.11±106.51	348.03±178.18	421.63±93.42	162.77±29.06	162.40±75.15
3	Dimethyl disulfide	624-92-0	Sulfur compounds	C2H6S2	94.2	129.751	235.2	14.583	RI + Drift time	Onion, garlic	391.91±64.05	436.66±104.10	590.98±58.29	164.68±28.63	202.09±102.11
4	Methional	3268-49-3	Sulfur compounds	C4H8OS	104.2	151.194	302.5	14.568	RI + Drift time	Potato, meaty	227.38±107.76	231.39±20.78	336.17±32.37	25.66±4.76	24.89±3.44
5	Dimethyl trisulfide	3658-80-8	Sulfur compounds	C2H6S3	126.3	123.549	213.6	12.448	RI + Drift time	Pungent, musty	12.90±0.88	54.66±3.11	99.06±0.89	183.65±22.61	174.83±13.99
6	Hexanal	66-25-1	Aldehydes	C6H12O	100.2	148.802	295.5	10.494	RI + Drift time	Green, fatty	283.96±46.28	443.24±69.17	557.41±57.82	50.52±8.81	65.39±12.52
7	Heptanal	111-71-7	Aldehydes	C7H14O	114.2	133.561	247.9	10.517	RI + Drift time	Oily, citrus	149.89±20.69	157.89±13.12	311.74±22.76	5504.89±131.93	2848.84±175.71
8	Octanal	124-13-0	Aldehydes	C8H16O	128.2	215.96	459.5	11.812	RI + Drift time	Citrus, fatty	76.97±28.57	110.88±15.81	141.72±13.09	1205.42±51.47	1816.00±127.37
9	Nonanal	124-19-6	Aldehydes	C9H18O	142.2	215.96	459.5	12.811	RI + Drift time	Rose, oily	172.76±40.94	183.13±9.83	211.86±18.95	986.39±12.16	1166.98±44.15
10	Benzaldehyde	100-52-7	Aldehydes	C7H6O	106.1	216.913	461.4	14.197	RI + Drift time	Almond, cherry	58.41±10.46	48.75±3.37	52.76±8.39	554.21±14.71	181.77±5.77
11	(E)-2-Hexenal	6728-26-3	Aldehydes	C6H10O	98.1	261.685	544	12.039	RI + Drift time	Green, apple	369.24±168.30	373.73±25.22	565.08±52.85	41.80±7.57	41.68±5.70
12	(E)-2-Heptenal	18829-55-5	Aldehydes	C7H12O	112.2	254.541	531.8	11.198	RI + Drift time	Fatty, oily	243.61±48.94	266.86±90.85	570.02±81.71	713.01±46.52	390.87±203.04
13	(E)-2-Octenal	2548-87-0	Aldehydes	C8H14O	126.2	239.299	504.6	12.266	RI + Drift time	Cucumber, nutty	165.10±152.50	61.42±6.24	67.51±4.22	1388.27±68.69	467.79±46.89
14	Phenylacetaldehyde	122-78-1	Aldehydes	C8H8O	120.2	235.965	498.4	12.857	RI + Drift time	Honey, floral	95.18±16.60	133.51±12.88	199.06±6.31	35.42±8.96	39.58±10.27
15	Butanal	123-72-8	Aldehydes	C4H8O	72.1	162.615	334.6	13.356	RI + Drift time	Pungent, cocoa	92.82±21.59	77.86±11.80	89.60±7.06	1464.58±111.44	1367.57±63.08
16	3-Methylbutanal	590-86-3	Aldehydes	C5H10O	86.1	137.371	260.3	11.903	RI + Drift time	Malty, chocolate	124.66±79.83	101.10±47.27	145.71±12.33	569.89±36.47	621.11±35.36
17	2-Methylbutanal	96-17-3	Aldehydes	C5H10O	86.1	141.169	272.3	11.548	RI + Drift time	Cocoa, coffee	97.56±16.58	135.29±16.18	197.45±9.49	35.92±7.68	38.95±8.52
18	Propanal	123-38-6	Aldehydes	C3H6O	58.1	234.587	495.9	16.154	RI + Drift time	Pungent, alcoholic	195.18±76.71	333.11±169.75	594.06±154.14	156.69±20.98	227.60±84.25
19	Decanal	112-31-2	Aldehydes	C10H20O	156.3	240	500	12	RI + Drift time	Citrus, waxy	287.44±2.25	603.75±41.40	1058.22±76.69	1588.43±93.11	1864.67±104.77
20	Pentanal	110-62-3	Aldehydes	C5H10O	86.1	311.704	621	10.942	RI + Drift time	Fermented, nutty	882.53±118.44	810.45±80.95	1000.12±124.71	867.42±81.25	933.80±57.16
21	(E)-2-Nonenal	18829-56-6	Aldehydes	C9H16O	140.2	349.317	671.1	11.058	RI + Drift time	Cucumber, fatty	871.24±32.40	963.07±125.61	776.95±50.35	858.50±49.60	898.62±97.32
22	1-Hexanol	111-27-3	Alcohols	C6H14O	102.2	138.291	263.3	12.819	RI + Drift time	Green, floral	39.51±13.01	41.32±2.41	39.38±7.09	773.41±13.25	444.42±1.25
23	1-Octen-3-ol	3391-86-4	Alcohols	C8H16O	128.2	99.738	119.4	12.274	RI + Drift time	Mushroom, musty	360.38±9.75	744.80±33.47	1361.56±137.22	1949.68±30.54	2386.02±135.98
24	1-Octanol	111-87-5	Alcohols	C8H18O	130.2	304.643	610.9	11.692	RI + Drift time	Citrus, oily	1319.22±502.93	175.79±23.70	148.62±11.93	1157.15±54.32	813.49±47.59
25	3-Octanol	589-98-0	Alcohols	C8H18O	130.2	417.812	749.9	13.279	RI + Drift time	Mushroom, earthy	264.08±41.23	235.75±16.30	341.63±2.68	390.48±8.76	604.31±21.83

26	1-Pentanol	71-41-0	Alcohols	C5H12O	88.1	453.905	786.4	12.343	RI + Drift time	Fermented, fruity	249.96±13.19	155.93±14.28	137.03±7.70	674.04±21.97	774.45±37.25
27	3-Methylbutan-1-ol	123-51-3	Alcohols	C5H12O	88.1	309.163	617.4	12.176	RI + Drift time	Whiskey, malty	1207.88±406.41	1336.38±128.78	950.94±96.90	523.30±31.81	348.20±9.30
28	2-Methylbutan-1-ol	137-32-6	Alcohols	C5H12O	88.1	308.419	616.3	12.946	RI + Drift time	Wine, fruity	623.55±846.18	239.05±29.85	1154.85±201.33	544.12±42.06	179.99±2.77
29	Ethanol	64-17-5	Alcohols	C2H6O	46.1	365.72	691.3	11.826	RI + Drift time	Alcohol, pungent	117.84±62.75	120.94±53.96	181.15±15.02	726.74±69.26	802.12±62.97
30	1-Propanol	71-23-8	Alcohols	C3H8O	60.1	361.999	686.8	13.821	RI + Drift time	Alcohol, fruity	3649.86±367.60	2941.04±735.14	2550.55±152.15	1019.28±49.68	886.81±66.29
31	2-Propanol	67-63-0	Alcohols	C3H8O	60.1	378.743	706.7	12.168	RI + Drift time	Solvent, cooling	414.47±151.00	649.89±56.15	894.40±17.07	162.58±4.76	101.84±10.53
32	(Z)-3-Hexen-1-ol	928-96-1	Alcohols	C6H12O	100.2	523.414	849.1	14.964	RI + Drift time	Green, leafy	258.45±106.48	423.05±204.62	743.05±218.91	201.56±33.04	279.77±98.33
33	(E)-2-Hexen-1-ol	928-95-0	Alcohols	C6H12O	100.2	560	880	12.5	RI + Drift time	Green, fruity	48.22±11.58	58.27±0.00	54.87±5.88	368.11±20.72	322.91±6.31
34	Ethyl acetate	141-78-6	Esters	C4H8O2	88.1	398.092	728.7	12.133	RI + Drift time	Fruity, solvent	731.17±61.53	605.59±5.27	759.94±14.52	870.10±42.39	951.67±34.57
35	Butyl acetate	123-86-4	Esters	C6H12O2	116.2	425.254	757.7	12.885	RI + Drift time	Banana, apple	177.33±99.31	146.53±9.85	199.43±4.16	32.18±7.97	34.43±9.58
36	Ethyl hexanoate	123-66-0	Esters	C8H16O2	144.2	425.584	758.1	12.209	RI + Drift time	Pineapple, fruity	611.44±361.13	1485.18±124.50	1874.08±183.96	40.49±0.00	45.83±4.82
37	Ethyl butyrate	105-54-4	Esters	C6H12O2	116.2	436.578	769.3	12.174	RI + Drift time	Pineapple, fruity	672.18±204.51	853.10±245.24	860.17±173.35	832.56±6.42	533.71±26.64
38	Ethyl propanoate	105-37-3	Esters	C5H10O2	102.1	259.425	540.2	14.528	RI + Drift time	Rum, fruity	126.91±12.17	140.79±14.36	176.09±33.68	572.34±44.45	635.87±21.13
39	Ethyl lactate	97-64-3	Esters	C5H10O3	118.1	503.078	831.7	12.202	RI + Drift time	Creamy, fruity	57.45±6.17	70.83±7.67	124.27±7.58	232.80±10.94	245.54±18.16
40	Methyl acetate	79-20-9	Esters	C3H6O2	74.1	256.2	530.5	12.485	RI + Drift time	Fruity, pleasant	116.56±11.29	76.24±8.61	98.39±10.18	192.86±7.69	146.03±5.88
41	2-Nonanone	821-55-6	Ketones	C9H18O	142.2	139.918	268.4	16.008	RI + Drift time	Fruity, cheesy	188.99±17.84	201.51±12.46	236.08±16.86	1004.25±64.85	1229.67±85.40
42	2-Heptanone	110-43-0	Ketones	C7H14O	114.2	504.122	832.6	11.301	RI + Drift time	Cheesy, fruity	749.11±44.05	657.02±28.85	823.38±18.06	947.51±25.29	1027.47±39.53
43	6-Methyl-5-hepten-2-one	110-93-0	Ketones	C8H14O	126.2	570.276	886.9	11.383	RI + Drift time	Citrus, herbal	193.14±114.79	154.22±12.27	210.27±13.67	35.32±8.14	37.68±9.72
44	Acetone	67-64-1	Ketones	C3H6O	58.1	664.35	954.1	11.467	RI + Drift time	Solvent, sweet	661.59±392.16	1563.34±79.45	2009.57±82.64	44.06±0.00	48.90±4.44
45	2-Butanone	78-93-3	Ketones	C4H8O	72.1	568.785	885.7	12.144	RI + Drift time	Solvent, sweet	287.79±105.35	531.33±71.17	570.46±64.01	35.48±9.99	59.84±9.02
46	2-Pentanone	107-87-9	Ketones	C5H10O	86.1	526.367	851.6	12.538	RI + Drift time	Fruity, ethereal	336.98±91.42	295.45±24.12	310.09±8.05	1616.04±54.72	685.69±10.98
47	2-Hexanone	591-78-6	Ketones	C6H12O	100.2	483.46	814.2	1.251	RI + Drift time	Fruity, green pepper	36.40±4.48	31.56±5.46	31.05±6.25	494.86±40.34	84.64±8.87
48	2,3-Pentanedione	600-14-6	Ketones	C5H8O2	100.1	654.598	947.6	12.594	RI + Drift time	Creamy, buttery	266.00±57.29	404.17±62.66	537.24±58.06	47.13±6.00	59.27±13.22
49	2-Pentylfuran	3777-69-3	Furans	C9H14O	138.2	598.527	908.2	13.919	RI + Drift time	Beany, green	766.15±354.21	697.38±544.14	836.66±397.97	372.46±85.40	458.85±274.62
50	Furfural	98-01-1	Furans	C5H4O2	96.1	669	957.4	1	RI + Drift time	Almond, bread	44.89±4.18	43.48±6.54	41.22±8.85	137.94±5.92	44.46±12.08
51	2-Methylfuran	534-22-5	Furans	C5H6O	82.1	707.256	981.6	13.242	RI + Drift time	Chocolate, nutty	665.17±179.61	680.20±344.02	843.15±246.80	327.29±75.38	336.90±161.30
52	2-Ethylfuran	3208-16-0	Furans	C6H8O	96.1	362.287	687.2	12.548	RI + Drift time	Roasted, coffee	780.43±148.30	876.46±217.15	1209.11±90.35	331.47±75.03	425.82±211.37
53	2-Methylpyrazine	109-08-0	Pyrazines	C5H6N2	94.1	381.094	709.5	12.856	RI + Drift time	Nutty, roasted	1830.01±243.49	1736.86±226.46	1837.13±78.24	1637.37±201.45	1728.85±198.62
54	2,5-Dimethylpyrazine	123-32-0	Pyrazines	C6H8N2	108.1	336.347	654.5	12.959	RI + Drift time	Roasted bean, cocoa	366.35±40.02	359.46±60.07	432.37±57.96	360.32±51.86	337.09±46.49
55	2,3,5-Trimethylpyrazine	14667-55-1	Pyrazines	C7H10N2	122.2	399.5	730.2	13.124	RI + Drift time	Roasted potato, baked	11.68±5.09	52.32±9.06	40.65±6.29	542.69±43.11	670.19±37.93

56	Hexanoic acid	142-62-1	Acids	C6H12O2	116.2	236.859	500.1	10.836	RI + Drift time	Sweaty, cheesy	222.61±29.53	234.22±17.27	286.62±14.86	1171.20±49.16	1401.32±39.80
57	Propanoic acid	79-09-4	Acids	C3H6O2	74.1	471.937	803.6	11.802	RI + Drift time	Sour, pungent	53.76±6.77	80.44±5.35	84.96±0.73	307.23±15.28	67.66±7.39
58	3-Methylbutanoic acid	503-74-2	Acids	C5H10O2	102.1	104.812	141.2	11.211	RI + Drift time	Sweaty, cheesy	219.35±33.36	240.69±7.31	273.22±22.41	1165.43±13.99	1407.13±53.55
59	Trimethylamine	75-50-3	Amines	C3H9N	59.1	754.276	1008.8	14.735	RI + Drift time	Fishy, ammoniacal	25.57±9.66	27.31±0.98	25.42±5.62	469.35±7.92	272.33±1.00
60	Dimethylamine	124-40-3	Amines	C2H7N	45.1	138.54	264	11.144	RI + Drift time	Fishy, putrid	26.14±2.23	23.78±1.85	24.73±5.35	544.86±22.29	801.90±67.43
61	Triethylamine	121-44-8	Amines	C6H15N	101.2	234.587	495.9	16.154	RI + Drift time	Ammoniacal, putrid	30.44±1.52	42.20±5.28	39.58±3.01	896.92±59.72	893.90±58.24
62	Diacetyl	431-03-8	Diketones	C4H6O2	86.1	171	357	2	RI + Drift time	Buttery, creamy	230.39±296.78	40.52±11.05	34.57±11.03	427.01±6.55	145.11±3.49

Table S2 Volatile compounds identified by HS-SPME-GC-MS in Thamnaconus modestus subjected to different processing methods.

Compound	CAS No.	Category	Molecular formula	MW	RT (min)	RI	Threshold (μg/kg)	Identification	Odor description	Raw (μg/kg)	Steamed (μg/kg)	Boiled (μg/kg)	Deep-fried (μg/kg)	Roasted (μg/kg)
Hexanal	66-25-1	Aldehydes	C6H12O	100.16	12.78	801	5	RI + MS + Standard	Green, fatty	117.37±10.00	283.25±28.93	194.47±38.00	632.76±54.02	505.56±19.21
Heptanal	111-71-7	Aldehydes	C7H14O	114.19	16.03	901	3	RI + MS + Standard	Fatty, citrus	34.22±3.73	93.87±13.46	65.42±3.05	154.23±9.14	214.53±23.79
Octanal	124-13-0	Aldehydes	C8H16O	128.22	19.28	1001	0.7	RI + MS + Standard	Citrus, fatty	22.55±2.48	67.19±8.25	45.63±7.86	154.33±6.61	126.85±16.51
Nonanal	124-19-6	Aldehydes	C9H18O	142.24	22.53	1101	1	RI + MS + Standard	Fatty, citrus, rose	36.56±3.92	125.51±6.63	73.96±2.58	234.74±60.20	225.95±35.92
Decanal	112-31-2	Aldehydes	C10H20O	156.27	25.78	1201	0.1	RI + MS + Standard	Citrus, fatty	7.57±0.80	21.74±1.02	14.66±1.01	54.61±1.56	44.59±3.97
Pentanal	110-62-3	Aldehydes	C5H10O	86.13	9.47	699	12	RI + MS + Standard	Almond, malty	55.61±5.90	131.22±8.05	110.94±8.51	318.33±41.21	266.13±10.51
3-Methylbutanal	590-86-3	Aldehydes	C5H10O	86.13	7.94	652	0.2	RI + MS + Standard	Malty, chocolate	0.00±0.00	17.71±2.29	12.65±1.64	64.84±6.45	106.14±20.95
(E)-2-Hexenal	6728-26-3	Aldehydes	C6H10O	98.14	14.54	855	17	RI + MS + Standard	Green, apple	29.74±2.56	0.00±0.00	0.00±0.00	125.03±11.40	82.20±9.62
(E)-2-Heptenal	18829-55-5	Aldehydes	C7H12O	112.17	17.88	958	13	RI + MS + Standard	Fatty, fishy	49.35±3.55	76.43±9.95	58.75±4.78	163.51±1.12	101.62±13.47
(E)-2-Octenal	2548-87-0	Aldehydes	C8H14O	126.2	21.2	1060	3	RI + MS + Standard	Fatty, nutty	14.80±1.15	0.00±0.00	30.99±5.25	94.59±5.05	76.63±11.94
(E)-2-Nonenal	18829-56-6	Aldehydes	C9H16O	140.23	24.48	1161	0.08	RI + MS + Standard	Fatty, cucumber	0.00±0.00	8.60±1.29	5.91±1.17	21.97±3.44	19.59±1.64
(E,E)-2,4-Heptadienal	4313-03-5	Aldehydes	C7H10O	110.16	19.74	1015	10	RI + MS + Standard	Fatty, deep-fried	0.00±0.00	16.55±2.94	9.63±2.22	110.48±8.18	72.22±4.08
(E,E)-2,4-Decadienal	25152-84-5	Aldehydes	C10H16O	152.24	29.52	1316	0.07	RI + MS + Standard	Oily, fishy	0.00±0.00	5.30±0.09	2.47±0.26	36.21±3.97	26.44±1.93
(E)-2-Decenal	3913-81-3	Aldehydes	C10H18O	154.25	27.77	1262	0.4	RI + MS + Standard	Fatty, citrus	0.00±0.00	6.30±1.20	4.10±0.64	28.70±1.42	23.79±0.72
Benzaldehyde	100-52-7	Aromatic aldehydes	C7H6O	106.12	18.02	962	350	RI + MS	Almond, cherry	20.94±1.89	54.05±6.44	34.12±8.73	191.96±10.51	222.90±27.55
Phenylacetaldehyde	122-78-1	Aromatic aldehydes	C8H8O	120.15	20.61	1042	4	RI + MS + Standard	Rose, honey	0.00±0.00	8.52±0.59	5.27±0.77	27.65±3.67	43.70±7.95
1-Octen-3-ol	3391-86-4	Alcohols	C8H16O	128.22	18.6	980	1	RI + MS + Standard	Mushroom, earthy	67.43±7.04	148.39±13.81	110.43±2.63	299.56±35.66	290.64±45.26
1-Penten-3-ol	616-25-1	Alcohols	C5H10O	86.13	8.91	682	400	RI + MS	Green, buttery	76.61±4.62	61.54±5.47	45.92±6.93	0.00±0.00	0.00±0.00
1-Hexanol	111-27-3	Alcohols	C6H14O	102.18	14.96	868	2500	RI + MS	Floral, green	91.70±22.12	0.00±0.00	0.00±0.00	42.04±2.14	31.19±1.18
1-Octanol	111-87-5	Alcohols	C8H18O	130.23	21.56	1071	110	RI + MS	Fatty, citrus	28.80±1.51	0.00±0.00	38.67±5.88	59.56±2.59	54.52±0.73
1-Pentanol	71-41-0	Alcohols	C5H12O	88.15	11.71	768	4000	RI + MS	Sweet, almond	109.00±11.06	62.32±8.21	56.33±6.88	0.00±0.00	0.00±0.00
1-Heptanol	111-70-6	Alcohols	C7H16O	116.2	18.24	969	425	RI + MS	Oily, green	23.48±1.51	0.00±0.00	0.00±0.00	47.76±2.81	39.97±4.69
2-Ethyl-1-hexanol	104-76-7	Alcohols	C8H18O	130.23	20.23	1030	270	RI + MS	Floral, citrus	16.13±1.67	20.96±2.10	16.95±0.34	32.78±5.55	27.28±1.90
Phenethyl alcohol	60-12-8	Aromatic alcohols	C8H10O	122.17	22.82	1110	1000	RI + MS	Rose, floral	0.00±0.00	13.56±2.21	7.37±0.23	33.58±2.54	53.08±0.92
2-Butanone	78-93-3	Ketones	C4H8O	72.11	6.09	595	50000	RI + MS	Sweet	43.54±7.23	56.23±0.93	48.30±6.19	83.79±5.96	66.03±12.49
2-Heptanone	110-43-0	Ketones	C7H14O	114.19	15.68	890	140	RI + MS	Creamy, fruity	17.33±2.81	35.99±0.93	26.02±2.70	91.94±4.86	116.82±10.01
2-Nonanone	821-55-6	Ketones	C9H18O	142.24	22.18	1090	41	RI + MS + Standard	Creamy, fruity	7.84±1.37	18.76±0.75	14.22±1.50	43.70±4.32	61.50±7.95

3-Octanone	106-68-3	Ketones	C8H16O	128.22	18.76	985	28	RI + MS + Standard	Herbal, mushroom	0.00±0.00	26.09±1.96	16.24±4.21	54.46±5.51	44.05±4.47
2-Octanone	111-13-7	Ketones	C8H16O	128.22	18.93	990	50	RI + MS	Creamy, fruity	0.00±0.00	14.13±0.44	10.08±0.16	41.86±6.45	25.97±4.71
6-Methyl-5-hepten-2-one	110-93-0	Ketones	C8H14O	126.2	18.76	985	50	RI + MS	Citrus, herbal	0.00±0.00	11.95±0.61	7.96±0.06	29.53±0.81	25.30±0.74
2,3-Butanedione	431-03-8	Diketones	C4H6O2	86.09	6.09	595	15	RI + MS + Standard	Buttery, creamy	0.00±0.00	22.06±0.74	17.96±4.03	59.15±10.38	86.29±9.01
2,3-Pentanedione	600-14-6	Diketones	C5H8O2	100.12	9.66	705	30	RI + MS + Standard	Buttery, caramel	5.31±0.88	14.71±1.53	10.43±1.02	45.79±4.48	54.82±5.05
3-Hydroxy-2-butanone	513-86-0	Hydroxy ketones	C4H8O2	88.11	9.82	710	800	RI + MS	Creamy, buttery	0.00±0.00	32.58±4.81	25.66±0.52	84.34±15.50	111.10±13.45
Dimethyl sulfide	75-18-3	Sulfur compounds	C2H6S	62.13	3.65	520	0.3	RI + MS + Standard	Cabbage, fishy	82.45±11.23	39.33±5.10	36.07±2.70	15.73±0.51	10.41±1.08
Dimethyl disulfide	624-92-0	Sulfur compounds	C2H6S2	94.2	10.96	745	0.16	RI + MS + Standard	Onion, fishy	51.54±5.32	24.51±5.48	21.28±0.55	8.31±1.05	4.88±0.88
Dimethyl trisulfide	3658-80-8	Sulfur compounds	C2H6S3	126.26	18.27	970	0.01	RI + MS + Standard	Onion, fishy	10.63±1.26	4.39±0.47	4.21±0.99	1.66±0.20	0.95±0.03
Methanethiol	74-93-1	Sulfur compounds	CH4S	48.11	2	460	0.02	RI + MS + Standard	Sulfurous, putrid	7.56±0.80	3.30±0.40	2.58±0.14	0.76±0.12	0.41±0.12
2-Pentylfuran	3777-69-3	Furans	C9H14O	138.21	18.99	992	6	RI + MS + Standard	Beany, green	0.00±0.00	44.12±7.90	31.19±0.50	171.61±8.03	173.66±13.34
Furfural	98-01-1	Furans	C5H4O2	96.08	13.79	832	3000	RI + MS	Caramel, almond	0.00±0.00	14.45±2.41	9.90±1.33	82.78±16.55	160.71±5.90
2-Ethylfuran	3208-16-0	Furans	C6H8O	96.13	9.6	703	2100	RI + MS	Sweet, caramel	0.00±0.00	9.96±0.40	8.00±0.40	44.90±6.04	74.63±10.55
2-Propylfuran	4229-91-8	Furans	C7H10O	110.16	13.4	820	nd	RI + MS	Green, beany	0.00±0.00	7.81±1.19	5.27±0.82	34.57±0.59	52.80±2.64
5-Methylfurfural	620-02-0	Furans	C6H6O2	110.11	18.11	965	16000	RI + MS	Caramel, almond	0.00±0.00	5.00±0.58	3.27±0.24	37.93±1.75	96.10±8.53
2-Acetylfuran	1192-62-7	Furans	C6H6O2	110.11	16.42	913	10000	RI + MS	Sweet, coffee	0.00±0.00	0.00±0.00	0.00±0.00	24.11±3.59	68.90±9.85
2-Methylpyrazine	109-08-0	Pyrazines	C5H6N2	94.12	13.46	822	60000	RI + MS	Nutty, roasted	0.00±0.00	0.00±0.00	0.00±0.00	43.45±2.73	121.53±11.39
2,5-Dimethylpyrazine	123-32-0	Pyrazines	C6H8N2	108.14	16.39	912	1800	RI + MS	Nutty, roasted	0.00±0.00	0.00±0.00	0.00±0.00	29.81±2.57	98.93±10.68
2,3,5-Trimethylpyrazine	14667-55-1	Pyrazines	C7H10N2	122.17	19.41	1005	400	RI + MS	Nutty, cocoa	0.00±0.00	0.00±0.00	0.00±0.00	19.74±1.36	70.26±12.35
2-Ethyl-3-methylpyrazine	15707-23-0	Pyrazines	C7H10N2	122.17	19.09	995	130	RI + MS	Nutty, roasted	0.00±0.00	0.00±0.00	0.00±0.00	13.16±2.91	45.85±4.98
2,6-Dimethylpyrazine	108-50-9	Pyrazines	C6H8N2	108.14	16.26	908	1500	RI + MS	Nutty, roasted	0.00±0.00	0.00±0.00	0.00±0.00	17.63±2.35	66.33±19.12
2,3,5,6-Tetramethylpyrazine	1124-11-4	Pyrazines	C8H12N2	136.2	21.85	1080	nd	RI + MS	Nutty, chocolate	0.00±0.00	0.00±0.00	0.00±0.00	7.60±0.94	34.82±2.86
Ethyl acetate	141-78-6	Esters	C4H8O2	88.11	6.64	612	5000	RI + MS	Fruity, sweet	35.87±3.00	20.22±2.44	13.20±1.39	9.71±1.43	8.10±1.35
Ethyl butyrate	105-54-4	Esters	C6H12O2	116.16	12.85	803	1	RI + MS + Standard	Fruity, pineapple	8.54±0.86	4.65±0.35	3.95±0.17	2.13±0.61	1.52±0.13
Ethyl hexanoate	123-66-0	Esters	C8H16O2	144.21	19.28	1001	1	RI + MS + Standard	Fruity, apple	4.80±0.53	3.18±0.49	1.97±0.23	1.04±0.12	0.82±0.04
Ethyl octanoate	106-32-1	Esters	C10H20O2	172.27	25.62	1196	2	RI + MS + Standard	Fruity, apricot	3.36±0.15	1.74±0.19	1.41±0.14	0.00±0.00	0.00±0.00
Tetradecane	629-59-4	Hydrocarbons	C14H30	198.39	32.25	1400	nd	RI + MS	Odorless	12.69±1.60	18.59±1.94	11.78±2.03	25.03±1.23	20.89±1.88
Pentadecane	629-62-9	Hydrocarbons	C15H32	212.42	35.5	1500	nd	RI + MS	Odorless	9.03±1.55	11.33±1.28	8.36±1.20	18.29±0.44	15.70±1.35
Hexadecane	544-76-3	Hydrocarbons	C16H34	226.45	38.75	1600	nd	RI + MS	Odorless	7.91±0.63	11.51±1.94	5.76±0.46	17.16±0.69	11.14±1.53
Toluene	108-88-3	Aromatic hydrocarbons	C7H8	92.14	11.78	770	nd	RI + MS	Aromatic	11.00±0.69	7.34±0.19	6.08±0.50	10.88±1.12	16.85±1.85

Styrene	100-42-5	Aromatic hydrocarbons	C8H8	104.15	15.77	893	nd	RI + MS	Sweet	4.80±0.49	4.05±0.44	2.99±0.36	7.97±0.58	9.64±0.96
2-Acetylthiazole	24295-03-2	Thiazoles	C5H5NOS	127.17	19.9	1020	10	RI + MS + Standard	Roasted meat, nutty	0.00±0.00	0.00±0.00	0.00±0.00	16.56±1.74	33.89±3.09
2-Acetylpyrrole	1072-83-9	Pyrroles	C6H7NO	109.13	21.52	1070	nd	RI + MS	Roasted, nutty	0.00±0.00	0.00±0.00	0.00±0.00	10.68±1.04	29.14±2.47
Trimethylamine	75-50-3	Amines	C3H9N	59.11	6.58	610	0.6	RI + MS + Standard	Fishy, ammonia	162.44±17.87	59.21±8.23	50.70±10.37	19.55±2.43	11.52±0.33
Acetic acid	64-19-7	Acids	C2H4O2	60.05	6.25	600	99000	RI + MS	Sour, vinegar	34.32±1.55	46.89±7.70	37.15±2.03	78.50±6.22	92.05±6.24
Butyric acid	107-92-6	Acids	C4H8O2	88.11	13.27	816	240	RI + MS	Rancid, cheesy	18.55±1.31	13.22±2.42	12.86±0.85	0.00±0.00	0.00±0.00
Hexanoic acid	142-62-1	Acids	C6H12O2	116.16	18.93	990	3000	RI + MS	Fatty, goaty	14.91±1.79	20.22±2.04	14.95±0.30	30.98±3.67	35.46±3.56
D-Limonene	5989-27-5	Terpenes	C10H16	136.24	20.19	1029	10	RI + MS	Citrus, fresh	8.60±0.47	5.61±0.39	5.53±0.97	0.00±0.00	0.00±0.00
2-Methylbutanal	96-17-3	Aldehydes	C5H10O	86.13	8.2	660	1	RI + MS + Standard	Malty, cocoa	0.00±0.00	20.60±2.01	15.48±0.41	55.86±1.73	84.08±9.86
2-Methyl-1-butanol	137-32-6	Alcohols	C5H12O	88.15	10.77	739	nd	RI + MS	Sweet, wine-like	0.00±0.00	18.76±0.81	11.94±0.09	8.58±2.07	5.32±0.66
2,4-Di-tert-butylphenol	96-76-4	Phenols	C14H22O	206.33	36.09	1518	nd	RI + MS	Odorless	0.00±0.00	0.00±0.00	0.00±0.00	44.73±5.17	0.00±0.00
(E)-2-Undecenal	53448-07-0	Aldehydes	C11H20O	168.28	31.27	1370	0.5	RI + MS + Standard	Fatty, fried	0.00±0.00	0.00±0.00	0.00±0.00	18.95±1.46	0.00±0.00
Dodecanal	112-54-9	Aldehydes	C12H24O	184.32	32.51	1408	0.1	RI + MS + Standard	Fatty, oily	0.00±0.00	0.00±0.00	0.00±0.00	13.12±0.68	0.00±0.00
2-Acetylpyrazine	22047-25-2	Pyrazines	C6H6N2O	122.13	21.2	1060	62	RI + MS	Roasted, popcorn	0.00±0.00	0.00±0.00	0.00±0.00	0.00±0.00	24.49±1.66
2-Acetyl-3-methylpyrazine	23787-80-6	Pyrazines	C7H8N2O	136.15	23.48	1130	nd	RI + MS	Roasted, nutty	0.00±0.00	0.00±0.00	0.00±0.00	0.00±0.00	19.39±5.29
Furfuryl alcohol	98-00-0	Furans	C5H6O2	98.1	14.99	869	nd	RI + MS	Caramel, roasted	0.00±0.00	0.00±0.00	16.84±2.41	0.00±0.00	45.74±5.84
(Z)-4-Heptenal	6728-31-0	Aldehydes	C7H12O	112.17	15.97	899	0.04	RI + MS + Standard	Fishy, creamy	15.81±1.74	0.00±0.00	0.00±0.00	0.00±0.00	0.00±0.00
1-Octen-3-one	4312-99-6	Ketones	C8H14O	126.2	18.57	979	0.005	RI + MS + Standard	Mushroom, metallic	3.05±0.79	0.00±0.00	0.00±0.00	0.00±0.00	0.00±0.00
(Z)-3-Hexenal	6789-80-6	Aldehydes	C6H10O	98.14	12.81	802	0.25	RI + MS + Standard	Green, leafy	11.70±0.67	0.00±0.00	0.00±0.00	0.00±0.00	0.00±0.00
(Z)-3-Hexen-1-ol	928-96-1	Alcohols	C6H12O	100.16	14.51	854	70	RI + MS	Green, leafy	20.32±1.09	0.00±0.00	0.00±0.00	0.00±0.00	0.00±0.00
1-Propanol	71-23-8	Alcohols	C3H8O	60.1	4.85	557	nd	RI + MS	Alcoholic, sweet	28.01±6.24	0.00±0.00	0.00±0.00	0.00±0.00	0.00±0.00
2-Butanol	78-92-2	Alcohols	C4H10O	74.12	5.92	590	nd	RI + MS	Sweet, alcoholic	21.64±2.10	0.00±0.00	0.00±0.00	0.00±0.00	0.00±0.00
3-Hexanone	589-38-8	Ketones	C6H12O	100.16	12.36	788	nd	RI + MS	Sweet, fruity	10.20±0.73	0.00±0.00	0.00±0.00	0.00±0.00	0.00±0.00
1-Nonen-3-ol	21964-44-3	Alcohols	C9H18O	142.24	21.69	1075	nd	RI + MS	Mushroom, earthy	8.36±0.83	0.00±0.00	0.00±0.00	0.00±0.00	0.00±0.00
(E)-2-Hexen-1-ol	928-95-0	Alcohols	C6H12O	100.16	14.77	862	nd	RI + MS	Green, fruity	16.09±0.38	0.00±0.00	0.00±0.00	0.00±0.00	0.00±0.00
2-Heptanol	543-49-7	Alcohols	C7H16O	116.2	15.84	895	nd	RI + MS	Mushroom, lemon	10.79±0.45	0.00±0.00	0.00±0.00	0.00±0.00	0.00±0.00
(Z)-1,5-Octadien-3-one	65767-22-8	Ketones	C8H12O	124.18	18.73	984	0.001	RI + MS + Standard	Geranium, metallic	2.25±0.09	0.00±0.00	0.00±0.00	0.00±0.00	0.00±0.00
(E,Z)-2,6-Nonadienal	557-48-2	Aldehydes	C9H14O	138.21	24.29	1155	0.01	RI + MS + Standard	Cucumber, watermelon	4.81±0.38	0.00±0.00	0.00±0.00	0.00±0.00	0.00±0.00
Indole	120-72-9	Pyrroles	C8H7N	117.15	28.84	1295	nd	RI + MS	Fecal, floral	12.08±0.25	0.00±0.00	0.00±0.00	0.00±0.00	0.00±0.00
3-Methyl-2-butenal	107-86-8	Aldehydes	C5H8O	84.12	12.16	782	nd	RI + MS	Fruity, almond	7.63±0.86	0.00±0.00	0.00±0.00	0.00±0.00	0.00±0.00

2-Methylpropanal	78-84-2	Aldehydes	C4H8O	72.11	4.79	555	1	RI + MS + Standard	Malty, green	15.04±0.56	0.00±0.00	0.00±0.00	0.00±0.00	0.00±0.00
1-Nonanol	143-08-8	Alcohols	C9H20O	144.26	24.81	1171	nd	RI + MS	Fatty, citrus	19.94±2.64	11.87±0.37	10.28±1.68	0.00±0.00	0.00±0.00
3-Methyl-1-butanol	123-51-3	Alcohols	C5H12O	88.15	10.8	740	250	RI + MS	Whiskey, malty	42.46±2.53	28.73±0.92	23.08±2.63	0.00±0.00	0.00±0.00
Ethanol	64-17-5	Alcohols	C2H6O	46.07	2	448	nd	RI + MS	Alcoholic, sweet	53.94±4.50	40.86±5.94	32.33±3.50	0.00±0.00	0.00±0.00
Ethyl propanoate	105-37-3	Esters	C5H10O2	102.13	9.99	715	10	RI + MS	Fruity, sweet	6.05±0.99	4.09±0.35	2.85±0.07	0.00±0.00	0.00±0.00
2-Methyl-1-propanol	78-83-1	Alcohols	C4H10O	74.12	7.06	625	nd	RI + MS	Wine-like, bitter	0.00±0.00	14.51±1.44	11.54±1.17	0.00±0.00	0.00±0.00
2-Propanol	67-63-0	Alcohols	C3H8O	60.1	3.33	510	nd	RI + MS	Alcoholic	0.00±0.00	23.65±2.70	19.58±2.86	0.00±0.00	0.00±0.00
2-Ethyl-5-methylpyrazine	13360-64-0	Pyrazines	C7H10N2	122.17	19.32	1002	nd	RI + MS	Nutty, roasted	0.00±0.00	0.00±0.00	0.00±0.00	15.77±1.19	39.48±3.63
Methional	3268-49-3	Sulfur compounds	C4H8OS	104.17	16.16	905	0.2	RI + MS + Standard	Cooked potato, meaty	0.00±0.00	0.00±0.00	0.00±0.00	12.79±1.20	26.81±2.48
2-Methylfuran	534-22-5	Furans	C5H6O	82.1	6.41	605	nd	RI + MS	Caramel, ethereal	0.00±0.00	0.00±0.00	0.00±0.00	29.40±2.45	55.98±0.83
Undecanal	112-44-7	Aldehydes	C11H22O	170.3	29.16	1305	0.1	RI + MS + Standard	Fatty, citrus	0.00±0.00	0.00±0.00	0.00±0.00	7.99±1.32	11.82±1.43
Undecane	1120-21-4	Hydrocarbons	C11H24	156.31	22.5	1100	nd	RI + MS	Gasoline	0.00±0.00	0.00±0.00	0.00±0.00	19.83±4.24	0.00±0.00
Dodecane	112-40-3	Hydrocarbons	C12H26	170.34	25.75	1200	nd	RI + MS	Gasoline	0.00±0.00	0.00±0.00	0.00±0.00	18.32±0.75	0.00±0.00
(E,E)-2,4-Nonadienal	5910-87-2	Aldehydes	C9H14O	138.21	26.17	1213	0.05	RI + MS + Standard	Oily, waxy	0.00±0.00	0.00±0.00	0.00±0.00	6.12±0.39	0.00±0.00
4-Vinylguaiacol	7786-61-0	Phenols	C9H10O2	150.18	29.42	1313	3	RI + MS + Standard	Smoky, clove	0.00±0.00	0.00±0.00	0.00±0.00	0.00±0.00	16.85±1.91
Pyridine	110-86-1	Pyridines	C5H5N	79.1	10.64	735	nd	RI + MS	Fishy, burnt	0.00±0.00	0.00±0.00	0.00±0.00	0.00±0.00	14.99±1.41
3-Ethyl-2,5-dimethylpyrazine	13360-65-1	Pyrazines	C8H12N2	136.2	21.79	1078	nd	RI + MS	Nutty, roasted	0.00±0.00	0.00±0.00	0.00±0.00	0.00±0.00	27.29±0.66
gamma-Butyrolactone	96-48-0	Esters	C4H6O2	86.09	16.49	915	nd	RI + MS	Caramel, creamy	0.00±0.00	11.45±0.57	0.00±0.00	0.00±0.00	0.00±0.00
2-Ethylpyrazine	13925-00-3	Pyrazines	C6H8N2	108.14	16.65	920	nd	RI + MS	Nutty, roasted	0.00±0.00	8.57±0.27	0.00±0.00	0.00±0.00	0.00±0.00
Methyl 2-methylbutyrate	868-57-5	Esters	C6H12O2	116.16	12.04	778	nd	RI + MS	Fruity, apple	0.00±0.00	11.35±1.70	0.00±0.00	0.00±0.00	0.00±0.00
gamma-Hexalactone	695-06-7	Esters	C6H10O2	114.14	21.04	1055	1600	RI + MS	Coconut, creamy	0.00±0.00	0.00±0.00	10.13±2.49	0.00±0.00	0.00±0.00
Ethyl 2-methylbutyrate	7452-79-1	Esters	C7H14O2	130.19	14.38	850	0.1	RI + MS + Standard	Fruity, apple	0.00±0.00	0.00±0.00	7.38±1.62	0.00±0.00	0.00±0.00
Pentanoic acid	109-52-4	Acids	C5H10O2	102.13	16	900	3000	RI + MS	Sweaty, sour	0.00±0.00	0.00±0.00	13.10±1.40	0.00±0.00	0.00±0.00
2-Methylbutyric acid	116-53-0	Acids	C5H10O2	102.13	14.7	860	22	RI + MS	Sweaty, cheesy	0.00±0.00	16.57±1.78	14.50±1.59	0.00±0.00	0.00±0.00
Isovaleric acid	503-74-2	Acids	C5H10O2	102.13	13.89	835	33	RI + MS	Sweaty, cheesy	0.00±0.00	14.78±0.85	11.80±0.65	0.00±0.00	0.00±0.00
2-Hexanone	591-78-6	Ketones	C6H12O	100.16	12.43	790	nd	RI + MS	Fruity, sweet	0.00±0.00	0.00±0.00	12.04±1.42	26.50±1.83	19.08±1.14
2-Pentanone	107-87-9	Ketones	C5H10O	86.13	9.04	686	nd	RI + MS	Sweet, fruity	0.00±0.00	0.00±0.00	0.00±0.00	20.74±4.06	29.81±1.34
2-Decanone	693-54-9	Ketones	C10H20O	156.27	25.46	1191	nd	RI + MS	Citrus, fatty	0.00±0.00	0.00±0.00	0.00±0.00	15.18±2.18	20.32±1.75
1-Hydroxy-2-propanone	116-09-6	Hydroxy ketones	C3H6O2	74.08	7.84	649	nd	RI + MS	Caramel, sweet	0.00±0.00	17.02±1.64	16.24±0.31	41.86±5.05	54.45±9.94
Octanoic acid	124-07-2	Acids	C8H16O2	144.21	25.1	1180	3000	RI + MS	Fatty, sweaty	0.00±0.00	12.97±1.19	9.06±0.53	25.39±4.17	30.13±5.31

2-Undecanone	112-12-9	Ketones	C11H22O	170.3	28.71	1291	7	RI + MS + Standard	Floral, fruity	0.00±0.00	4.69±0.30	3.57±0.46	14.18±1.76	18.19±1.95
3-Pentanone	96-22-0	Ketones	C5H10O	86.13	9.34	695	nd	RI + MS	Sweet, fruity	0.00±0.00	17.83±2.26	13.17±2.12	38.19±5.02	27.50±3.13
Tridecane	629-50-5	Hydrocarbons	C13H28	184.37	29	1300	nd	RI + MS	Odorless	0.00±0.00	7.74±0.97	6.51±0.88	14.21±1.61	12.39±1.18
(Z)-2-Heptenal	57266-86-1	Aldehydes	C7H12O	112.17	17.62	950	nd	RI + MS	Fatty, green	0.00±0.00	28.78±4.44	20.44±2.05	72.52±8.25	52.02±1.30
(E)-2-Octen-1-ol	18409-17-1	Alcohols	C8H16O	128.22	21.43	1067	nd	RI + MS	Green, waxy	0.00±0.00	13.67±0.61	12.80±0.03	36.68±4.83	25.63±2.64
(E)-4-Heptenal	25524-95-2	Aldehydes	C7H12O	112.17	15.9	897	nd	RI + MS	Creamy, biscuit	0.00±0.00	9.98±0.16	8.49±0.50	29.99±2.35	34.42±2.88
(E)-2-Nonen-1-ol	31502-14-4	Alcohols	C9H18O	142.24	24.77	1170	nd	RI + MS	Fatty, cucumber	0.00±0.00	7.79±0.44	6.28±1.24	18.24±1.79	23.43±2.58
Propanoic acid	79-09-4	Acids	C3H6O2	74.08	9.5	700	20000	RI + MS	Sour, pungent	0.00±0.00	19.14±1.33	17.93±1.89	35.06±2.32	44.55±4.45
2-Tridecanone	593-08-8	Ketones	C13H26O	198.35	35.34	1495	nd	RI + MS	Fatty, fruity	0.00±0.00	4.67±0.57	4.06±0.31	9.81±2.56	15.01±2.21
2-Dodecenal	4826-62-4	Aldehydes	C12H22O	182.31	34.39	1466	nd	RI + MS	Fatty, citrus	0.00±0.00	4.47±0.67	3.36±0.12	14.99±2.56	16.47±1.62
Heptadecane	629-78-7	Hydrocarbons	C17H36	240.47	42	1700	nd	RI + MS	Odorless	0.00±0.00	5.53±0.32	4.51±0.81	11.12±1.61	8.61±1.08
2-Butylfuran	4466-24-4	Furans	C8H12O	124.18	15.64	889	nd	RI + MS	Green, fatty	0.00±0.00	10.32±1.03	7.92±1.32	41.37±4.47	45.56±1.33

Table S3 Key aroma compounds screened by odor activity value (OAV) in Thamnaconus modestus subjected to different processing methods.

Name	CAS	Raw(μg/kg)	Steamed(μg/kg)	Boiled(μg/kg)	Deep-fried(μg/kg)	Roasted(μg/kg)
Hexanal	66-25-1	117.37	283.25	194.47	632.76	505.56
Heptanal	111-71-7	34.22	93.87	65.42	154.23	214.53
Octanal	124-13-0	22.55	67.19	45.63	154.33	126.85
Nonanal	124-19-6	36.56	125.51	73.96	234.74	225.95
Decanal	112-31-2	7.57	21.74	14.66	54.61	44.59
Pentanal	110-62-3	55.61	131.22	110.94	318.33	266.13
3-Methylbutanal	590-86-3	0	17.71	12.65	64.84	106.14
(E)-2-Hexenal	6728-26-3	29.74	0	0	125.03	82.2
(E)-2-Heptenal	18829-55-5	49.35	76.43	58.75	163.51	101.62
(E)-2-Octenal	2548-87-0	14.8	0	30.99	94.59	76.63
(E)-2-Nonenal	18829-56-6	0	8.6	5.91	21.97	19.59
(E,E)-2,4-Heptadienal	4313-03-5	0	16.55	9.63	110.48	72.22
(E,E)-2,4-Decadienal	25152-84-5	0	5.3	2.47	36.21	26.44
(E)-2-Decenal	3913-81-3	0	6.3	4.1	28.7	23.79
Phenylacetaldehyde	122-78-1	0	8.52	5.27	27.65	43.7
1-Octen-3-ol	3391-86-4	67.43	148.39	110.43	299.56	290.64
2-Nonanone	821-55-6	7.84	18.76	14.22	43.7	61.5
3-Octanone	106-68-3	0	26.09	16.24	54.46	44.05
2,3-Butanedione	431-03-8	0	22.06	17.96	59.15	86.29
2,3-Pentanedione	600-14-6	5.31	14.71	10.43	45.79	54.82
Dimethyl sulfide	75-18-3	82.45	39.33	36.07	15.73	10.41
Dimethyl disulfide	624-92-0	51.54	24.51	21.28	8.31	4.88
Dimethyl trisulfide	3658-80-8	10.63	4.39	4.21	1.66	0.95
Methanethiol	74-93-1	7.56	3.3	2.58	0.76	0.41
2-Pentylfuran	3777-69-3	0	44.12	31.19	171.61	173.66
Ethyl butyrate	105-54-4	8.54	4.65	3.95	2.13	1.52
Ethyl hexanoate	123-66-0	4.8	3.18	1.97	1.04	0.82

Ethyl octanoate	106-32-1	3.36	1.74	1.41	0	0
2-Acetylthiazole	24295-03-2	0	0	0	16.56	33.89
Trimethylamine	75-50-3	162.44	59.21	50.7	19.55	11.52
2-Methylbutanal	96-17-3	0	20.6	15.48	55.86	84.08
(E)-2-Undecenal	53448-07-0	0	0	0	18.95	0
Dodecanal	112-54-9	0	0	0	13.12	0
(Z)-4-Heptenal	6728-31-0	15.81	0	0	0	0
1-Octen-3-one	4312-99-6	3.05	0	0	0	0
(Z)-3-Hexenal	6789-80-6	11.7	0	0	0	0
(Z)-1,5-Octadien-3-one	65767-22-8	2.25	0	0	0	0
(E,Z)-2,6-Nonadienal	557-48-2	4.81	0	0	0	0
2-Methylpropanal	78-84-2	15.04	0	0	0	0
Methional	3268-49-3	0	0	0	12.79	26.81
Undecanal	112-44-7	0	0	0	7.99	11.82
(E,E)-2,4-Nonadienal	5910-87-2	0	0	0	6.12	0
4-Vinylguaiacol	7786-61-0	0	0	0	0	16.85
Ethyl 2-methylbutyrate	7452-79-1	0	0	7.38	0	0
2-Undecanone	112-12-9	0	4.69	3.57	14.18	18.19

Table S4 Standard curve parameters for external standard quantification.

Name	CAS	Standard curve equation	R^2	Limit of detection LOD(μg/kg)	Limit of quantification LOQ (μg/kg)	Slope	Intercept
Hexanal	66-25-1	y = 2904.58x + 4739.94	0.99967	15.3057	46.381	2904.58	4739.94
Heptanal	111-71-7	y = 4452.95x -1667.21	0.999909	2.7321	8.279	4452.95	-1667.21
Octanal	124-13-0	y = 4032.59x -914.84	0.999953	1.4092	4.2703	4032.59	-914.84
Nonanal	124-19-6	y = 3303.09x + 1273.71	0.999983	1.2764	3.8678	3303.09	1273.71
Decanal	112-31-2	y = 4364.76x -606.97	0.9998	1.0338	3.1328	4364.76	-606.97
Pentanal	110-62-3	y = 1928.55x + 987.23	0.999989	1.405	4.2577	1928.55	987.23
3-Methylbutanal	590-86-3	y = 1461.78x -56.67	0.999934	1.1572	3.5066	1461.78	-56.67
(E)-2-Hexenal	6728-26-3	y = 4260.40x -761.78	0.999965	0.9792	2.9673	4260.4	-761.78
(E)-2-Heptenal	18829-55-5	y = 2526.23x + 305.98	0.999939	1.6812	5.0947	2526.23	305.98
(E)-2-Octenal	2548-87-0	y = 3672.41x -567.93	0.999986	0.4807	1.4566	3672.41	-567.93
(E)-2-Nonenal	18829-56-6	y = 1249.98x -53.91	0.999908	0.2788	0.8449	1249.98	-53.91
(E,E)-2,4-Heptadienal	4313-03-5	y = 4597.78x -76.94	0.999975	0.7411	2.2458	4597.78	-76.94
(E,E)-2,4-Decadienal	25152-84-5	y = 2181.74x + 33.33	0.999985	0.1888	0.5722	2181.74	33.33
(E)-2-Decenal	3913-81-3	y = 1944.68x -1.64	0.99999	0.1219	0.3695	1944.68	-1.64
Phenylacetaldehyde	122-78-1	y = 3368.69x + 484.39	0.999937	0.4643	1.407	3368.69	484.39
1-Octen-3-ol	3391-86-4	y = 3230.85x -857.25	0.999951	2.797	8.4756	3230.85	-857.25
2-Nonanone	821-55-6	y = 4427.16x + 383.06	0.999919	0.7403	2.2434	4427.16	383.06
3-Octanone	106-68-3	y = 1021.67x + 145.62	0.999951	0.5008	1.5176	1021.67	145.62
2,3-Butanedione	431-03-8	y = 1434.59x -78.82	0.999952	0.7923	2.4011	1434.59	-78.82
2,3-Pentanedione	600-14-6	y = 2145.62x + 145.33	0.999959	0.4704	1.4253	2145.62	145.33
Dimethyl sulfide	75-18-3	y = 1689.08x + 77.82	0.999978	0.5141	1.558	1689.08	77.82
Dimethyl disulfide	624-92-0	y = 4003.78x + 62.56	0.999975	0.344	1.0426	4003.78	62.56
Dimethyl trisulfide	3658-80-8	y = 1480.16x -4.88	0.999829	0.1866	0.5654	1480.16	-4.88
Methanethiol	74-93-1	y = 4999.12x -16.53	0.999976	0.0502	0.152	4999.12	-16.53
2-Pentylfuran	3777-69-3	y = 4033.04x + 1021.19	0.99989	2.4289	7.3603	4033.04	1021.19
Ethyl butyrate	105-54-4	y = 3788.56x + 20.17	0.999981	0.0499	0.1512	3788.56	20.17
Ethyl hexanoate	123-66-0	y = 4320.79x -19.42	0.999985	0.0247	0.0747	4320.79	-19.42
Ethyl octanoate	106-32-1	y = 3669.61x + 18.02	0.999905	0.0427	0.1293	3669.61	18.02

2-Acetylthiazole	24295-03-2	y = 1916.32x -83.63	0.999977	0.2115		0.6409	1916.32	-83.63
Trimethylamine	75-50-3	y = 3694.48x -2.56	0.999973	1.1337		3.4354	3694.48	-2.56
2-Methylbutanal	96-17-3	y = 3660.48x + 635.10	0.999978	0.5251		1.5913	3660.48	635.1
(E)-2-Undecenal	53448-07-0	y = 3121.56x -190.75	0.999823	0.3122		0.9461	3121.56	-190.75
Dodecanal	112-54-9	y = 1582.30x + 84.84	0.999798	0.2313		0.7009	1582.3	84.84
(Z)-4-Heptenal	6728-31-0	y = 1139.71x + 18.01	0.999771	0.2968		0.8994	1139.71	18.01
1-Octen-3-one	4312-99-6	y = 1823.24x + 0.72	0.999937	0.0299		0.0907	1823.24	0.72
(Z)-3-Hexenal	6789-80-6	y = 4483.29x -0.06	0.999978	0.0685		0.2075	4483.29	-0.06
(Z)-1,5-Octadien-3-one	65767-22-8	y = 3096.39x + 13.46	0.999981	0.0123		0.0372	3096.39	13.46
(E,Z)-2,6-Nonadienal	557-48-2	y = 3397.55x -50.89	0.999922	0.0526		0.1593	3397.55	-50.89
2-Methylpropanal	78-84-2	y = 3273.76x -62.63	0.999973	0.0972		0.2944	3273.76	-62.63
Methional	3268-49-3	y = 3871.43x -102.17	0.999808	0.482		1.4606	3871.43	-102.17
Undecanal	112-44-7	y = 4370.34x + 112.06	0.999988	0.0529		0.1602	4370.34	112.06
(E,E)-2,4-Nonadienal	5910-87-2	y = 3286.60x -20.98	0.99994	0.0588		0.1783	3286.6	-20.98
4-Vinylguaiacol	7786-61-0	y = 2216.37x -62.63	0.999971	0.1118		0.3387	2216.37	-62.63
Ethyl 2-methylbutyrate	7452-79-1	y = 3317.96x -65.83	0.999933	0.0746		0.2261	3317.96	-65.83
2-Undecanone	112-12-9	y = 1097.22x + 6.66	0.999986	0.0891		0.2701	1097.22	6.66
